# Metronomic cyclophosphamide activation of anti-tumor immunity: tumor model, mouse host, and drug schedule dependence of gene responses and their upstream regulators

**DOI:** 10.1186/s12885-016-2597-2

**Published:** 2016-08-11

**Authors:** Junjie Wu, Marie Jordan, David J. Waxman

**Affiliations:** Division of Cell and Molecular Biology, Department of Biology and Bioinformatics Program, Boston University, 5 Cummington Mall, Boston, MA 02215 USA

**Keywords:** Metronomic cyclophosphamide, Immune responsiveness, Cd8^+^ T cells, NK cells, RNA-seq, Upstream regulator, Drug schedule

## Abstract

**Background:**

Cyclophosphamide (CPA) can activate immunogenic tumor cell death, which induces immune-based tumor ablation and long-term anti-tumor immunity in a syngeneic C57BL/6 (B6) mouse GL261 glioma model when CPA is given on a 6-day repeating metronomic schedule (CPA/6d). In contrast, we find that two other syngeneic B6 mouse tumors, LLC lung carcinoma and B16F10 melanoma, do not exhibit these drug-induced immune responses despite their intrinsic sensitivity to CPA cytotoxicity.

**Methods:**

To elucidate underlying mechanisms, we investigated gene expression and molecular pathway changes associated with the disparate immune responsiveness of these tumors to CPA/6d treatment.

**Results:**

Global transcriptome analysis indicated substantial elevation of basal GL261 immune infiltration and strong CPA/6d activation of GL261 immune stimulatory pathways and their upstream regulators, but without preferential depletion of negative immune regulators compared to LLC and B16F10 tumors. In LLC tumors, where CPA/6d treatment is shown to be anti-angiogenic, CPA/6d suppressed VEGFA target genes and down regulated cell adhesion and leukocyte transendothelial migration genes. In GL261 tumors implanted in adaptive immune-deficient scid mice, where CPA/6d-induced GL261 regression is incomplete and late tumor growth rebound can occur, T cell receptor signaling and certain cytokine-cytokine receptor responses seen in B6 mice were deficient. Extending the CPA treatment interval from 6 to 9 days (CPA/9d) − which results in a strong but transient natural killer cell response followed by early tumor growth rebound − induced fewer cytokines and increased expression of drug metabolism genes.

**Conclusions:**

These findings elucidate molecular response pathways activated by intermittent metronomic CPA treatment and identify deficiencies that characterize immune-unresponsive tumor models and drug schedules.

**Electronic supplementary material:**

The online version of this article (doi:10.1186/s12885-016-2597-2) contains supplementary material, which is available to authorized users.

## Background

Certain cytotoxic anti-cancer drugs, including doxorubicin, oxaliplatin, and cyclophosphamide (CPA), can activate immunogenic tumor cell death, triggering robust anti-tumor immune responses [1, 2]. Hallmarks of immunogenic cell death [1, 3] include exposure of calreticulin on the tumor cell surface, which serves as an “eat-me” signal for dendritic cells [4] and macrophages [5], and HSP90 exposure, which facilitates dendritic cell-tumor cell adhesion and stimulates dendritic cell maturation [6]. Release of the alarmin molecule HMGB1 into the extracellular matrix activates toll-like receptors and stimulates dendritic cell antigen presentation and IL1β production, leading to CD8 T cell activation [7, 8], while ATP released from apoptotic tumor cells can activate dendritic cells [9]. Less well defined are the downstream anti-tumor immune responses induced by these initial immunogenic cell death events, as well as the factors that determine whether a cytotoxic drug intrinsically capable of eliciting immunogenic cell death will actually do so, and in a way that induces a strong anti-tumor immune response.

CPA is a bifunctional alkylating agent widely used for cancer treatment [10]. CPA can induce immunogenic tumor cell death and activate robust immune responses in several tumor models [11–13]. In gliomas, CPA induces major tumor regression and activates robust anti-tumor immune responses when given on a metronomic schedule [14, 15] that is intermittent, involving repeated dosing every 6 days (CPA/6d), as seen in both scid (adaptive immune-deficient) mice and in fully immune competent C57BL/6 (B6) mice [16–20]. Glioma regression achieved in scid mice may be followed by late tumor growth rebound [17, 18], whereas regression is sustained in a fully immune competent, syngeneic B6 mouse model and leads to long-term tumor-specific immunity [20]. Several interferons were identified as major upstream regulators of the immune responses in CPA/6d-treated U251 (human) and 9L (rat) gliomas implanted in scid mice [21]. Tumor-associated gene responses identified in these xenograft models include induction of many cytokines, chemokines, and immune regulatory genes linked to innate immune cell recruitment and tumor regression, as well as tumor escape [21]. It is unclear, however, which pathways and mechanisms dominate the response to CPA/6d treatment in an immune competent mouse host. Also unknown is whether strong anti-tumor immune responses are activated by the CPA/6d regimen in other, non-glioma models that show strong intrinsic sensitivity to CPA cytotoxicity.

Here, we use global transcriptomic profiling by RNA-seq to investigate the molecular signaling pathways associated with the immune response of GL261 gliomas to intermittent metronomic CPA treatment in a syngeneic, immune competent B6 mouse host. The profile of gene expression changes seen in GL261 gliomas, which show strong immune responsiveness to CPA/6d treatment, is compared to the changes seen in two other B6 mouse syngeneic tumor models that are intrinsically sensitive to CPA cytotoxicity, LLC Lewis lung carcinoma and B16F10 melanoma, but which, we show here, do not undergo CPA-induced tumor regression or mount a strong anti-tumor immune response. We also compare gene responses induced by CPA/6d treatment of GL261 tumors implanted in scid mice (GL261(scid)) to those induced in GL261 tumors in the fully immune competent B6 mouse model (GL261(B6)). Finally, we compare gene responses of CPA/6d-treated GL261(scid) tumors to those induced when CPA is given on a 9-day repeating metronomic schedule (CPA/9d), where tumor-infiltrating natural killer (NK) cell and other innate immune responses are initially very strong but are not sustained, and where early resumption of robust tumor growth occurs, despite continued CPA treatment [18]. Molecular pathways associated with tumor responsiveness to metronomic CPA treatment are identified along with upstream regulators (UPRs) predicted by Ingenuity Pathway Analysis (IPA). Our findings give new insights into the genes and pathways that characterize the wide range of immune responsiveness to anti-cancer drug treatment seen in different tumor models and help elucidate the impact of drug scheduling and the adaptive immune system on tumor responses to immunogenic metronomic chemotherapy.

## Methods

### Tumor cell lines, mouse tumors, and treatments

Mouse tumor cell lines syngeneic in B6 mice were authenticated by and obtained from Developmental Therapeutics Program Tumor Repository (National Cancer Institute, Frederick, MD) (GL261 glioma) and ATCC (Manassas, VA) (LLC Lewis lung carcinoma (ATCC® CRL-1642™) and B16F10 melanoma (ATCC® CRL-6475™)). RNA-seq analysis (below) of the X-chromosome gene Xist and the Y-chromosome genes Ddx3y and Eif2s3y indicated that GL261 and B16F10 tumor cells are male, and LLC tumor cells are female. Cells were grown at 37 °C in a humidified 5 % CO_2_ atmosphere in RPMI-1640 culture medium containing 10 % fetal bovine serum, 100 units/ml penicillin and 100 μg/ml streptomycin. Tumor cells were assayed for their intrinsic sensitivity to activated CPA using 4-hydroperoxy-CPA in a 4-day growth inhibition assay [22]. Six-week-old (20–23 g) male B6 mice and 6-week-old (26–28 g) male ICR/Fox Chase immune deficient scid mice (Taconic Farms, Germantown, NY) were housed and treated using protocols specifically reviewed for ethics and approved by the Boston University Institutional Animal Care and Use Committee. GL261 glioma cells (4 × 10^6^) were implanted by s.c. injection on the posterior flanks of B6 mice (GL261(B6) model) [20] or scid mice (GL261(scid) model) [18] in 0.2 ml serum-free RPMI per site using a U-100 insulin syringe and a 28.5 gauge needle (BD Biosciences, Cat.# 329461). B16F10 melanoma cells (1 × 10^6^) or LLC Lewis lung carcinoma cells (2 × 10^6^) were implanted by s.c. injection on the posterior flanks of B6 mice in the same manner. Tumor areas (length × width) were measured twice weekly using Vernier calipers (VWR International, Cat.# 62379-531) and tumor volumes were calculated: Vol = (π/6)*(L*W)^3/2^. Tumor volumes (~500–750 mm^3^, mean values) were normalized to a value of 100 % on the first day of CPA treatment (t = 0 days) to control for differences in initial tumor size. This enabled us to reach statistical significance with fewer mice [20], in accordance with our Institutional Animal Care and Use Committee guidelines. Mouse body weights were measured at least twice a week and normalized in the same manner. Tumor-bearing mice were treated every 6 days with CPA at 140 mg/kg-body weight (CPA/6d) [16, 17]. CPA was administered as a monohydrate (Cat. # C0768, Sigma-Aldrich, St. Louis, MO) dissolved in sterile PBS, with the CPA doses reported here based on the non-hydrated molecular weight of 261. Where indicated, mice bearing GL261(scid) tumors were treated with same dose of CPA, but given on a 9 day repeating schedule (CPA/9d). In all cases, qPCR and RNA-seq analysis were carried out using tumor tissue from untreated mice (control group) and from CPA-treated mice collected 6 days after the last CPA treatment. Thus, we monitored gene expression at a consistent point in time for all tumor samples, including CPA/6d-treated vs. CPA/9d-treated GL261 tumors. Additional file 1: Table S1 shows the number of mice included in each treatment group.

### Tumor blood vessel immunostaining

Cryosections prepared from LLC tumors were mounted on slides and immunostained with antibody to mouse CD31 (BD Pharmingen, Cat.# 557355, 1:1000 dilution) to visualize tumor blood vessels. Cyosections were hydrated in 1x PBS, fixed in 3.2 % paraformaldehyde for 15 min, permeabilized with 1 % Triton X-100 on ice for 5 min, treated with 3 % H_2_O_2_ for 5 min, and blocked for 20 min in 4 % normal rabbit serum in PBS (blocking solution) containing avidin (Vector Labs, avidin/biotin blocking kit, Cat.# SP-2001) at 4 drops/ml. Slides were incubated for 1 h at room temperature with anti-CD31 antibody (1:1000 dilution in blocking solution containing biotin (Vector Labs) at 4 drops/ml), followed by 1 h incubation at room temperature with biotinylated rabbit anti-rat secondary antibody (Vector labs, Cat.# 4000, 1:200 dilution in blocking solution), followed by Vectastain ABC reagent (Vector labs, Cat.# PK4000) for 30 min, and VIP (Vector labs, Cat. # SK-4600) color development for 40 s. Each step, above, was followed by a PBS wash. Color development was terminated by two 5 min washes in distilled water, followed by a 5 min rinse in tap water. Slides were dehydrated in 95 % ethanol for 2 min twice, followed by 100 % ethanol for 2 min twice, xylene for 3 min twice, and Vectamount mounting (Vector Labs, Cat.# H-5000). Slides were dried overnight in a fume hood and imaged with an Olympus FSX100 microscope at 4.2x magnification. Images were converted to gray scale for quantification using NIH image J software (http://imagej.nih.gov/ij/).

### Gene expression analysis by qPCR and RNA-seq

Extraction of total RNA from individual tumors using Trizol reagent, and qPCR analysis of relative RNA levels for immune cell marker genes were performed as described [16]. Tumor RNA samples having Agilent Bioanalyzer RIN values > 7 were grouped into two separate pools (biological replicates) for each treatment group and used for RNA-seq library preparation and high throughput sequencing, as detailed in the legend to Additional file 1: Table S1. For GL261 tumors implanted in scid mice, RNA-seq libraries were prepared using the Illumina TruSeq® mRNA library Prep kit (Cat# RS-122-2101). RNA-seq libraries were prepared from all other tumor RNA samples using NebNext® Ultra Directional RNA Library Prep kit for Illumina® (New England Biolabs, Ipswich, MA; Cat# E7420). NEBNext® Multiplex Oligos for Illumina® (New England Biolabs, Cat# E7335S) were used for sample multiplexing. Agencourt AMPure XP beads (Beckman Coulter, Cat# A63880) were used for size selection and purification. Library quality and insert size distribution were assessed using the Agilent Bioanalyzer DNA high sensitivity chip kit (Agilent Technologies, Cat# 5067-4627). Samples were subject to Illumina sequencing using a HiSeq instrument generating either 50 or 68 nt single-end reads. All raw and processed sequencing data are available under accession number GSE71491 at GEO (http://www.ncbi.nlm.nih.gov/geo/); further details are shown in Additional file 1: Table S1 legend. Sequence reads were demultiplexed and then aligned to the mouse genome (build mm9; NCBI 37) using Tophat (version 2.0.13) [23, 24]. Differential expression analysis for RefSeq genes was conducted using the Bioconductor package DESeq (version 1.18.0) [25]. CPA-induced gene responses meeting the cutoff values of |fold-change| (FC) > 2 and *p* < 0.001 for each tumor model and treatment condition are shown in Additional file 1: Table S1A-F and summarized in Additional file 2: Figure S1.

### Upstream regulator analysis

A combined list of genes that were either up regulated or down regulated by CPA treatment at |FC| > 2 and *p* < 0.001 was uploaded together with the corresponding gene expression FC values for Ingenuity Pathway Analysis (IPA) (www.ingenuity.com/products/ipa). The Upstream Analysis module of IPA was used to identify upstream regulators (UPRs), their predicted activation Z-scores and bias-corrected Z-scores, targeted molecules in each dataset, *p*-values of overlap between targeted genes and UPR-regulated genes in the IPA database, and the associated mechanistic networks. Individual UPRs were identified as being “Activated” or “Inhibited” as predicted by IPA. Since our goal was to identify endogenous master UPRs induced by metronomic CPA treatment, we excluded all UPRs that are classified by IPA as chemicals, except endogenous mammalian chemicals. We also excluded group UPRs that duplicate individual constituent UPRs. When two UPRs with same name were identified, e.g., one from mouse another from human, the UPR with higher activation score was retained. The resultant full UPR lists are shown in Additional file 3: Table S2A-E. Overall results obtained using UPR analysis are summarized in Additional file 2: Figure S2.

To increase the stringency of UPR identification, UPRs identified by IPA using IPA’s default conditions were filtered by applying the following conditions: *p*-value of overlap < 0.0001, number of targeted genes > 10, and absolute values of the predicted activation Z-score and bias-corrected activation Z-score both > 2 (stringent UPRs; Additional file 2: Figure S3). The following criteria were applied to assess the uniqueness of each stringent UPR to a given tumor model, as outlined in Additional file 2: Figure S4. A stringent UPR identified in tumor model A was designated unique to tumor model A, as compared to tumor model B, if it met *either* of the following two conditions: (1) the UPR was absent from the listing of UPRs generated by IPA under default conditions for tumor model B; (2) |activation Z-score| and |bias-corrected Z-score| for the UPR are both > 2 in tumor model B, but show the opposite activation state, i.e., Activated in one tumor model vs. Inhibited in the other tumor model. UPRs that met either of the following two criteria were considered as *candidate* unique UPRs for tumor model A: (1) |activation Z-score| and |bias-corrected Z-score| for the UPR are both < 2 in tumor model B; or (2) either |activation Z-score| or |bias-corrected Z-score| for the UPR in tumor model B, but not both, is > 2, and is in the opposite direction as for the UPR in tumor model A. The *p*-value of overlap was then used to determine the uniqueness of each candidate unique UPR, following the last three decision tree steps in Additional file 2: Figure S4. Thus, if the *p*-value of overlap for a given candidate UPR was ≥ 0.001 in tumor model B, the UPR was designated unique to tumor model A. Alternatively, if the *p*-value of overlap of the candidate UPR in tumor model B was < 0.001 but ≥ 0.0001, and if it was > 100-fold higher than the *p*-value for overlap of that UPR in tumor model A, then the UPR was designated unique to tumor model A. However, if the *p*-value of overlap of a given candidate UPR was < 0.0001 in tumor model B, the UPR was not considered unique to tumor model A, despite its having an |activation Z-score| or a |bias-corrected Z-score| < 2 in tumor model B, and even if its *p*-value of overlap was > 100-fold higher than that of the UPR in the model A (Additional file 2: Figure S4).

The numbers of stringent UPRs identified in each tumor model (Additional file 4: Table S3A-E) and each tumor model comparison (which identify either common or unique UPRs for each model; Additional file 4: Table S3F-K) are summarized in Additional file 2: Figure S2. To identify CPA-induced UPRs unique to GL261(scid) tumors as compared to GL261(B6) tumors, we compared the full set of UPRs associated with CPA-induced gene responses in GL261(B6) tumors (Additional file 3: Table S2A) to the set of 179 stringent UPRs common to the responses of GL261(scid) tumors to CPA/6d and CPA/9d treatment (Additional file 4: Table S3F), as outlined in Additional file 2: Figure S2 (*right*). This approach was based on the high overall similarity of gene responses (77 % similarity, Additional file 1: Table S1F) and stringent UPRs (Additional file 4: Table S3F vs. Additional file 4: Tables S3D-E) between the CPA/6d and CPA/9d treatments, both of which effected strong tumor regression when the tumors were collected 6 days after the second CPA injection. When assessing the uniqueness of the stringent UPRs identified in the GL261(B6) tumor model (Additional file 4: Table S3A) as compared to the GL261(scid) model, we considered the full set of UPRs derived from CPA/6d-treated GL261(scid) tumors (Additional file 3: Table S2D; see Additional file 2: Figure S2).

### KEGG pathway analysis

Genes that were up or down regulated significantly by CPA treatment at |(FC)| > 2 and *p* < 0.001 were input as separate gene lists for analysis using DAVID Bioinformatics Resources 6.7 with default parameters (http://david.abcc.ncifcrf.gov/tools.jsp) to identify KEGG pathways, as well as DAVID functional annotation clusters (DAVID clusters) enriched in each set of CPA-regulated genes. KEGG pathways with *p*-values < 0.05 and DAVID clusters with enrichment score > 1.3 were deemed significant (Additional file 5: Table S4). KEGG pathways specific to one tumor model or treatment condition were identified from the sets of genes that showed a significant response to CPA in a given direction (e.g., up regulation) in tumor model or treatment condition A but not in tumor model/treatment condition B, as follows. Genes that showed significant up regulation by CPA at |FC| > 2 and *p* < 0.001 in tumor model A and significant down regulation in tumor model B were considered specific response genes for both A and B. Other tumor model A-specific response genes were those showing at least a 2-fold greater response in tumor model A than tumor model B, as follows. We first identified all genes showing a significant response to CPA (|FC| > 2 and *p* < 0.001) in model A but not model B, and then removed all genes for which 0.5 ≤ expression ratio-Tumor model A/expression ratio-Tumor model B ≤ 2. The combined set of all tumor model A-specific up regulated genes (and similarly for down regulated genes) was then used to identify KEGG pathways specific to tumor model A, as compared to tumor model B, as outlined in Additional file 2: Figure S5 and S6.

When comparing KEGG pathways activated by the CPA/6d and CPA/9d schedules, where a limited number of tumor model-specific genes were identified, we relaxed the threshold for a difference in expression ratios between tumor models from >2-fold to >1.33-fold when inputting genes for pathway analysis. In an alternative approach, we reduced the significance filter for genes under consideration to |FC| > 1.5 and *p* < 0.001, as specified in the text. When comparing KEGG pathways activated in GL261(scid) vs. GL261(B6) tumors, we increased the robustness of the analysis by considering those GL261(scid) genes showing a significant response to both CPA/6d and CPA/9d (Additional file 1: Table S1F).

### Immunosuppressive factors

A list of 124 immunosuppressive genes was compiled from the Gene Ontology term “Negative regulation of immune response” (GO:0050777), which included 196 human and 153 mouse genes. Human gene symbol were converted to mouse gene symbols using MammalHom (http://depts.washington.edu/l2l/mammalhom.html) or by manually checking the NCBI Gene database (http://www.ncbi.nlm.nih.gov/gene/) where no mouse genes were identified by MammalHom. Genes redundant between human and mouse, and isoforms within a given species were removed. The resultant list of 124 negative regulators of immune response (Additional file 6: Table S5A) was used to identify immune suppressive factors that may contribute to the differential CPA responsiveness between tumor models, as well as differences in response between B6 and scid mouse host, and between the CPA/6d and CPA/9d schedules.

## Results

### Metronomic CPA does not activate robust immune cell recruitment or induce tumor regression in LLC and B16F10 tumors

**Fig. 1:**
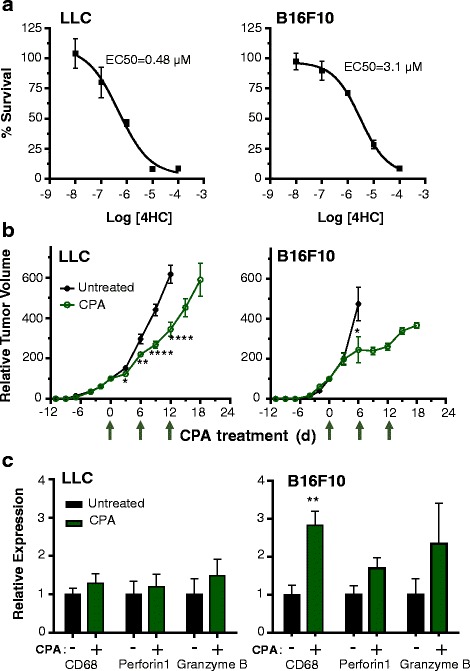
Intrinsic sensitivity to activated CPA, anti-tumor activity, and immune cell recruitment/activation in metronomic CPA-treated B16-F10 and LLC tumors. **a**, Sensitivity of LLC and B16F10 tumor cell lines to 4-hydroperoxy-CPA in cell culture, determined using a 4-day growth inhibition assay. EC50, effective concentration for 50 % growth inhibition. EC50 for 4-hydroperoxy-CPA-treated GL261 cells, 0.15 μM (data not shown). **b**, In vivo tumor growth profiles for LLC and B16F10 tumors in response to treatment with 140 mg/kg CPA on treatment days 0, 6, and 12 (*arrows* along X-axis). **c**, qPCR analysis of the indicated immune markers in CPA-treated and untreated LLC and B16F10 tumors (shown in **b**) implanted s.c. in B6 mice. Data in **a** is representative of *n* = 5 culture wells per data point, data in **b** is based on mean ± SE for *n* = 10–14 tumors per group, and data in **c** based on *n* = 4–5 tumors per group. *, *p* < 0.05 by two-tailed *t*-test

Metronomic CPA treatment on a 6-day repeating schedule (CPA/6d) induces a complete, immune cell-dependent regression of GL261 tumors implanted in immune competent C57BL/6 mice (GL261(B6) tumor model) and activates long-term tumor-specific immunity [20]. We investigated two other B6 syngeneic tumor cell lines, LLC Lewis lung carcinoma and B16F10 melanoma, both of which are intrinsically sensitive to the cytotoxic effects of 4-hydroxy-CPA, the activated form of CPA (Fig. 1a). In contrast to GL261 tumors [20], LLC and B16F10 tumors showed only minor (LLC) or moderate (B16F10) tumor growth delay in response to metronomic CPA treatment (Fig. 1b). Differences in tumor growth rate do not account for the differences of CPA/6d-induced growth inhibition of LLC and B16F10 tumors compared to each other and compared to GL261 tumors (data not shown). Rather, analysis of marker genes for macrophages (CD68) and cytotoxic effectors of natural killer and T cells (perforin1, granzyme B) indicated that metronomic CPA induced weak (B16F10) or no increases (LCC) in immune cell marker genes (Fig. 1c), in contrast to the strong increases in all three immune markers following CPA/6d treatment of GL261 tumors in the same B6 mouse model [20]. LLC and B16F10 tumors were therefore designated metronomic CPA immune unresponsive (Table 1).

**Table 1:** Tumor models, mouse hosts, CPA schedules, gene responses and UPR analysis. RNA-seq was performed on two replicated RNA pools for each condition (Additional file 1: Table S1 legend)

	Responsive tumors				Unresponsive tumors	
Tumor	GL261				LLC	B16F10
Mouse host	B6	scid	scid	scid	B6	B6
CPA schedule (days)	6	6	9	6 and 9^a^	6	6
Tumor responses to CPA treatment	Complete regression	Major regression with late rebound	Major regression with early rebound	–	Minor growth delay	Moderate growth delay
Up regulated genes	2119	2574	2713	2352	151	663
Down regulated genes	809	1250	1564	1176	70	394
Stringent Upstream Regulators (UPRs)	180	210	218	179	6	93

Shown here is a summary of tumor responses to drug treatment, for GL261(B6) [20], GL261(scid) [18], LLC and B16F10 tumors (Fig. 1), the number of genes up or down regulated at |FC| > 2 and *p* < 0.001 (Additional file 1: Table S1), and the number of significant UPRs (Additional file 4: Table S3) after filtering of the full set of UPRs output by IPA (Additional file 3: Table S2)

^a^, Number of commonly regulated genes and UPRs responding in common between GL261(scid) tumors treated with CPA/6d and GL261(scid) tumors treated with CPA/9d

### Gene expression changes and their upstream regulators (UPRs) in responsive vs. unresponsive tumor models

Global transcriptional profiling by RNA-seq was used to further characterize immune-based gene responses in metronomic CPA-treated GL261(B6) tumors and to elucidate the extent of immune unresponsiveness of LLC and B16F10 tumors and its underlying mechanisms. We identified 2119 up regulated genes and 809 down regulated genes in CPA/6d-treated GL261(B6) tumors (significance cutoff values |FC| > 2 and *p* < 0.001; Table 1, Additional file 1: Table S1A). Many fewer genes (663 up regulated, 394 down regulated genes) responded to CPA in B16F10 tumors, and even fewer gene responses were seen in LLC tumors (151 up regulated, 70 down regulated genes; Table 1, Additional file 1: Table S1B-C), which are the most intrinsically sensitive to activated CPA but showed the weakest anti-tumor and immune responses (Fig. 1). Analysis of the regulated gene sets in each tumor model identified upstream regulators (UPRs), which were predicted to be either activated or inhibited by metronomic CPA based on the direction of response of their downstream target genes. The full sets of UPRs associated with the CPA-responsive genes in each tumor model (Additional file 3: Table S2A-C) were used to identify top UPRs (Table 1, stringent UPRs; Additional file 4: Table S3A-C) by applying more stringent selection criteria (Additional file 2: Figures S2, S3, S4). Stringent UPRs that were uniquely associated with a given tumor model or that were common between tumors models were also identified.

47 out of 180 stringent UPRs associated with GL261(B6) tumor responses to CPA were unique to GL261(B6) tumors as compared to both unresponsive tumor models (Additional file 4: Table S3G). These 47 UPRs encompass four categories (Table 2): 1) Factors that facilitate tumor regression by immune-mediated mechanisms (23 activated UPRs that activate immune responses, including HMGB1, an immunogenic cell death marker [26], and 3 inhibited UPRs that inhibit immune responses), or by inhibiting tumor cell survival (6 inhibited UPRs that promote tumor cell survival); 2) Factors that counteract tumor regression by inhibiting immune responses (activated PTGS2 [27]) or by promoting cell survival (activated UPRs FN1 [28] and FGFR2 [29]); 3) Factors that are either positive or negative immune response modulators, depending on the cell context (10 UPRs); 4) Glioma cell lineage-related factors (activated UPRs SIM1 [30] and PAX7 [31]).

**Table 2:** Unique UPRs induced by CPA in GL261(B6) compared to LLC and B16F10 tumors. Shown are the 47 UPRs unique to CPA-treated GL261(B6) tumors identified in Additional file 4: Table S3G, classified into 4 categories based on their functions. Group 1 UPRs are expected to contribute to the anti-tumor response, group 2 UPRs counter the anti-tumor response, and the actions of group 3 UPRs depend on cell context. Only two of the UPRs are associated with the glioma-specific lineage of GL261 tumors (group 4)

Category	Reported function	Predicted activation state	Molecule type	Upstream regulator
1. Facilitate tumor regression by immune-mediated mechanisms or by inhibiting tumor cell survival	Activate immune responses	Activated	Cytokine	IL12 (complex), IL7,IL12A,IL12B,CCL11
			Enzyme	TRAF6
			Kinase	MAPK8,MAPKAPK2,MAP3K14,RIPK2
			Other	MOG,TAC1
			Transcription regulator	TBX21,HMGB1,IRF6,HOXA7
			Transmembrane receptor	TLR2,TYROBP,CD2,CD14,OLR1,CD86,BTNL2
	Inhibit immune responses	Inhibited	Transcription regulator	PRDM1
			Neurohormone	CORT
			Phosphatase	DUSP1
	Promote tumor cell survival	Inhibited	Enzyme	SCD
			Growth factor	WISP2
			Kinase	PRKAA1
			Mature microRNA	miR-155-5p (miRNAs w/seed UAAUGCU)
			Transcription regulator	MAX,BCL3
2. Counter tumor regression	Inhibit immune respones	Activated	Enzyme	PTGS2
	Promote tumor cell survival	Activated	Enzyme	FN1
			Kinase	FGFR2
3. Postive or negatve regulator of immune response, depending on cell context	Activate or inhibit immunity	Activated	Cytokine	CSF2,CXCL8,PF4
			G-protein coupled receptor	CCR5
			Apoptotic factor	TRADD
	Activate or inhibit immunity	Inhibited	Enzyme	TAB1
			Kinase	MTOR
			Other	PTX3
			Transcription regulator	IRF4
			Transporter	APOA1
4. Glioma cell lineage	Brain development	Activated	Transcription regulator	SIM1,PAX7

**Fig. 2:**
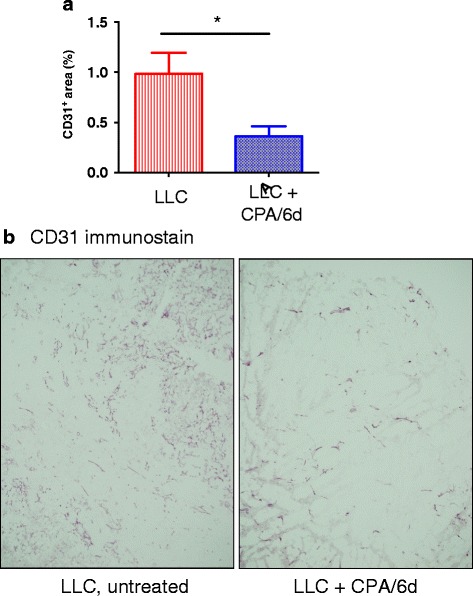
Impact of CPA/6d treatment on LLC tumor microvessel density. Immunohistochemcal staining of blood vessel marker CD31 in LLC tumor sections from untreated or CPA/6d-treated tumors, 6 days after the third CPA treatment. **a**, relative CD31 staining intensity, mean ± SE for *n* = 8 tumors/group; *p < 0.05 by two-tailed *t*-test. **b**, representative figure for each tumor group shown in (**a**)

VEGFA was identified as an activated UPR in CPA-treated GL261(B6) tumors (Additional file 4: Table S3A), consistent with the requirement for VEGFA signaling via VEGFR2 for CPA/6d to induce immune cell recruitment in responding gliomas [16, 19]. In contrast, VEGFA was a uniquely inhibited UPR in CPA-treated LLC tumors (Additional file 4: Table S3B), suggesting that CPA/6d inhibits VEGFA-dependent angiogenesis in this tumor model. VEGFA was not a significant UPR in B16F10 tumors. The predicted UPR activity of VEGFA in LLC tumors was verified experimentally by the significant reduction in LLC tumor microvessel density following CPA/6d treatment (Fig. 2). We also identified six UPRs unique to CPA-treated B16F10 tumors vs. GL261(B6) tumors that promote tumor cell survival or tissue repair and may contribute to B16F10 tumor unresponsiveness: activated UPRs KDM5B [32], RBL2 [33] and SPARC [34–36]; and inhibited UPRs FOXM1 [37], CD24 [38, 39] and CSF2 [40]) (Additional file 4: Table S3C). CSF2, which stimulates intra-tumoral dendritic cell expansion and induces significant CD4+ and CD8+ T cell anti-tumor immune responses [41, 42] was the top uniquely activated UPR (most significant UPR, based on *p*-value of overlap) in CPA-treated GL261 tumors (Table 2, Additional file 4: Table S3G).

59 stringent UPRs were common to GL261(B6) and B16F10 tumors (Additional file 4: Table S3H). Of these, the top (most significant) UPRs include IFNB1, IFNG, IL1B, IL6, TGFB1, TNF and TP53, which are activated UPRs, and MYC, which is inhibited. IFNB1, IFNG and IL6 were also identified as highly significant activated UPRs in studies of CPA/6d-treated U251 and 9L tumors in scid mice [21]. However, the fact that these same UPRs are also activated in the CPA/6d immune unresponsive B16F10 tumors indicates that activation of these UPRs alone is insufficient to induce a robust downstream anti-tumor immune response. Three top GL261(B6) and B16F10 common UPRs have a more significant *p*-value of overlap in B16F10 tumors than in GL261(B6) tumors (activated UPRs CDKN2A and IRF7; inhibited UPR TBX2; Additional file 4: Table S3H), suggesting they might contribute to the reduced responsiveness of B16F10 tumors. The activation of three stringent UPRs related to DNA damage pathways [43–45] in both GL261 and LLC tumors (CHUK/IKBKA, IKBKB and JUN) or in all three tumor models (CHUK, IKBKB) (Additional file 4: Table S3A-C) may reflect the cytotoxic responses common to CPA treatment in all three tumor models.

### KEGG pathways activated in responsive and unresponsive tumor models

Immune response-related pathways were most highly enriched in the 2119 genes up regulated by CPA/6d in GL261(B6) tumors. The top 24 pathways (*p* < 0.0001) can be classified into three groups (Table 3A; full pathway listing in Additional file 5: Table S4A): 1) immune stimulatory signaling; 2) immune effector activation, including NK cell-mediated cytotoxicity, T cell, and B cell signaling; and 3) inflammatory pathways activated in immune-related diseases. The top KEGG pathways enriched among the 809 genes down regulated in GL261(B6) tumors are primarily involved in tumor cell essential survival functions (Table 3B; Additional file 5: Table S4A), consistent with the strong tumor regression induced by CPA/6d treatment.

**Table 3:** KEGG pathways responded to CPA in GL261(B6) tumors

A. Top up regulated pathways				
		Gene Count	%	*P*-Value
Immuno-stimulatory signaling pathways	mmu04060:Cytokine-cytokine receptor interaction	105	5.05	1.36E-30
	mmu04142:Lysosome	50	2.41	4.61E-14
	mmu04514:Cell adhesion molecules (CAMs)	55	2.65	5.33E-12
	mmu04062:Chemokine signaling pathway	59	2.84	7.45E-11
	mmu04630:Jak-STAT signaling pathway	50	2.41	1.51E-09
	mmu04620:Toll-like receptor signaling pathway	38	1.83	1.71E-09
	mmu04512:ECM-receptor interaction	34	1.64	2.09E-09
	mmu04621:NOD-like receptor signaling pathway	27	1.30	2.96E-08
	mmu04612:Antigen processing and presentation	32	1.54	4.18E-07
	mmu04510:Focal adhesion	53	2.55	9.28E-07
	mmu04210:Apoptosis	29	1.40	5.42E-06
	mmu04610:Complement and coagulation cascades	25	1.20	2.82E-05
	mmu05340:Primary immunodeficiency	16	0.77	2.88E-05
	mmu04670:Leukocyte transendothelial migration	33	1.59	7.23E-05
Immune effector activation pathway	mmu04650:Natural killer cell mediated cytotoxicity	56	2.69	7.15E-18
	mmu04660:T cell receptor signaling pathway	39	1.88	1.13E-07
	mmu04662:B cell receptor signaling pathway	30	1.44	2.18E-07
Pathways in immune related disease	mmu04640:Hematopoietic cell lineage	45	2.17	1.28E-17
	mmu05332:Graft-versus-host disease	31	1.49	3.57E-12
	mmu04940:Type I diabetes mellitus	28	1.35	9.06E-09
	mmu05330:Allograft rejection	26	1.25	2.82E-08
	mmu05416:Viral myocarditis	31	1.49	3.05E-06
	mmu04672:Intestinal immune network for IgA production	21	1.01	1.16E-05
	mmu05320:Autoimmune thyroid disease	25	1.20	1.30E-05
	mmu05322:Systemic lupus erythematosus	29	1.40	1.65E-04
B. Top down regulated pathways				
Tumor cell essential function pathways	mmu00100: Steroid biosynthesis	10	1.28	1.45E-09
	mmu00900: Terpenoid backbone biosynthesis	8	1.02	1.80E-07
	mmu03030: DNA replication	10	1.28	2.41E-06
	mmu00240: Pyrimidine metabolism	14	1.79	2.95E-05
	mmu04110: Cell cycle	16	2.04	4.15E-05

KEGG pathways enriched at *p* < 0.0001 in the sets of genes up regulated (A) or down regulated (B) by CPA in GL261(B6) tumors (Additional file 1: Table S1A). The complete set of KEGG pathways identified is shown in Additional file 5: Table S4A. Related KEGG pathways were grouped into categories, as shown. Count, number of input genes associated with the particular pathway; %, percentage of input genes associated with the particular term as a percent of total input genes. P Value, modified Fisher Exact *P*-Value output from David analysis

In LLC tumors, the 151 genes up regulated by CPA/6d (Table 1) are most highly enriched in KEGG pathways related to focal adhesion, ECM-receptor interaction and complement and coagulation cascades (Additional file 5: Table S4B), which include genes related to immune stimulatory signaling. The 70 CPA-down regulated LLC genes are enriched for cell adhesion molecules and leukocyte transendothelial migration (Additional file 5: Table S4B). The latter two pathways, which are up regulated in GL261(B6) tumors (Table 3A), are critical for tumor infiltration by immune cells [46]; their down regulation in LLC tumors may contribute to LLC low immune responsiveness.

The 663 genes up regulated in CPA/6d-treated B16F10 tumors (Table 1) are enriched for p53 signaling, which may regulate tumor cell response to CPA at the level of DNA damage response and immune response, as well as 10 other KEGG pathways related to immune stimulatory signaling or immune-related diseases: lysosomes [47], complement and coagulation cascades, systemic lupus erythematosus, cytokine-cytokine receptor interactions, prion disease [48], JAK-STAT signaling, chemokine signaling, antigen processing and presentation, cell adhesion molecules, and ECM-receptor interaction (Additional file 5: Table S4C). However, in contrast to GL261(B6) tumors, immune effector activation pathways were not enriched in the metronomic CPA responses in B16F10 tumors, which may explain why activation of the above 10 pathways is not sufficient for a robust anti-tumor immune response in B16F10 tumors. The set of 394 down regulated B16F10 genes (Table 1) is enriched in tumor growth-related pathways, such as cell cycle, DNA replication, serine and threonine metabolism, one carbon pool by folate, steroid biosynthesis, terpenoid backbone biosynthesis, and pyrimidine metabolism (Additional file 5: Table S4C), consistent with the moderate B16F10 tumor growth delay induced by CPA treatment (Fig. 1). The down regulation of serine and threonine metabolism may lead to a decrease in glutathione levels [49, 50] and thereby sensitize B16F10 tumor cells to the cytotoxicity of CPA.

### Gene pathways predictive of differential responsiveness to CPA in untreated tumors

LLC, B16F10 and GL261 tumor cells all show substantial intrinsic sensitivity to activated CPA cytotoxicity (Fig. 1a) at concentrations well below those reached in tumor-bearing mice given CPA/6d treatments [17]. Accordingly, we hypothesize that their differential responsiveness to CPA in vivo is not due to differences in intrinsic CPA sensitivity, but rather, reflects the distinct interactions of each tumor cell line with host stromal cells. To identify genes and pathways active in untreated tumors that are associated with, and may be predictive of, this differential responsiveness to metronomic CPA treatment, we compared the transcriptional profiles of untreated GL261 tumors to those of untreated LLC and B16F10 tumors. We identified 1348 genes showing significantly higher basal expression and 438 genes showing significantly lower basal expression in the responsive tumors than in both unresponsive tumor models (Additional file 7: Tables S7A-B, Additional file 2: Figure S7). Unexpectedly, the genes more highly expressed in the responsive tumors were enriched for immune-related signaling pathways, including immune effector signaling, immune stimulatory signaling, and immune disease (Additional file 8: Table S8A). The strongest enrichment was for cell adhesion genes, many of which are involved in antigen processing and presentation and other immune responses. Only two of the enriched pathways (axon guidance and prion diseases) were related to the neuronal cell lineage of GL261 tumors. Thus, basal immune activity is higher in the responsive tumors and may positively impact responsiveness to CPA-induced immunity.

Strikingly, 44 % of the 1348 genes with higher basal expression in GL261(B6) tumors showed a significant change in expression following CPA/6d treatment, a 3.5-fold enrichment when compared to the 12.6 % response rate for all genes (Additional file 7: Table S7C, legend). Thus, the pathways dysregulated by CPA/6d are already primed to be differentially expressed in untreated GL261 tumors. Immune activation-related pathways were significantly enriched in the genes with higher basal expression that were up regulated by CPA (Additional file 8: Table S8B), while glycine, serine and threonine metabolism and focal adhesion were significantly enriched in the CPA down regulated genes (Additional file 8: Table S8C). The down regulation of glycine, serine and threonine metabolism may reduce the anti-oxidative capacity of GL261 tumor cells and sensitize them to CPA cytotoxicity [49]. Genes related to glial cells and neuron establishment were also significantly enriched in the CPA down regulated gene set (Additional file 8: Table S8D).

The 438 genes showing significantly lower basal expression in responsive tumors compared to both unresponsive tumor models (Additional file 7: Table S7B) are most highly enriched for glutathione metabolism and other metabolic pathways (Additional file 8: Table S8E). These pathways include glutathione S-transferases GSTM1 and GSTP1, which are associated with resistance to CPA [51, 52]. Further, lower basal GL261(B6) expression was seen for glycolysis/gluconeogenesis and for the lysosome pathway, which degrades/recycles macromolecules via endocytosis, phagocytosis and autophagy, suggesting the responsive tumors have a low metabolic rate [53]. 147 of the 438 genes showing lower basal expression in GL261(B6) tumors were up regulated by CPA/6d treatment; only one gene was down regulated (Additional file 7: Table S7D). These 147 genes are enriched for lysosome pathway, blood vessel morphogenesis, pleckstrin homology-type, glycoprotein, and endosome/cytoplasmic membrane-bounded vesicle (Additional file 8: Table S8F). Up regulation of the endosome-lysosome pathway may enhance immunogenic tumor cell death by increasing digestion, processing and presentation of tumor antigens [54]. Up regulation of blood vessel morphogenesis may increase tumor uptake of active CPA metabolites and immune cell recruitment into tumors [55–57].

### Negative regulators of immune response

CPA alteration of immune suppressive factors in the tumor microenvironment could contribute to differences in chemotherapy-induced immune responses between tumor models. To determine whether CPA depletes such immune suppressive factors, we compared the full set of CPA-responsive genes in each tumor model with a set of 124 negative regulators of immune response (Additional file 6: Table S5B; Additional file 8: Figure S8). Surprisingly, in GL261(B6) tumors, only one of the 124 genes was down regulated by CPA/6d treatment (*CR2*, FC = −2.1), while 57 genes were up regulated (Additional file 6: Table S5C). Further, CPA up regulated only 4 of the 124 negative regulators in LLC tumors and 17 in B16F10 tumors (Additional file 6: Table S5D, Additional file 6: Table S5E). One negative regulator of immune response was down regulated by CPA treatment in LLC tumors (*Hmox1*, FC = −2.2) and no negative regulators of immune response were down regulated in B16F10 tumors. No negative regulators of immune response were uniquely up regulated by CPA (>2-fold difference) in the two unresponsive tumor models as compared to GL261(B6) tumors. Consistently, 48 negative regulators of immune response were expressed at a higher level and none was expressed at a lower level in CPA-treated GL261(B6) tumors than in CPA-treated LLC and B16F10 tumors (Additional file 6: Table S5F). Further, we identified 20 negative immune regulators expressed at a significantly *higher* basal level in untreated GL261 tumors than in untreated LLC and B16F10 tumors (Additional file 6: Table S5G), whereas only one negative regulator, HMOX1, showed significantly lower basal expression in GL261 tumors. Thus, the lack of robust immune responses in CPA-treated LLC and B16F10 tumors cannot be attributed to a more immune suppressive microenvironment, either basally or following CPA treatment. Further, the strong immune response in CPA-treated GL261(B6) tumors apparently occurs in spite of elevated basal immunity.

### Differential GL261 tumor responses in scid vs. immune competent B6 mouse host

We sought to identify genes and signaling pathways that underlie the more complete and durable anti-tumor responses that CPA/6d induces in GL261 tumors implanted in B6 mice [20] as compared to adaptive immune system deficient scid mice [18]. Large numbers of genes showed common responses to metronomic CPA in both mouse models (Additional file 9: Table S10A, B, Additional file 2: Figure S1B), with enrichment for KEGG pathways similar to those described above for the B6 model alone (Additional file 10: Table S11A-C). Many fewer genes (Additional file 9: Table S10C-F; Additional file 2: Figure S6) and KEGG pathways (Additional file 10: Table S11D, E) showed significant differential responses between B6 and scid mouse hosts, consistent with the overall similarity of innate immune and anti-tumor responses seen in CPA/6d-treated GL261(scid) and GL261(B6) tumors [16, 18, 20].

The top three KEGG pathways enriched in the set of 130 genes up regulated by CPA/6d specifically in GL261(B6) compared to GL261(scid) tumors are immune-related: cytokine-cytokine receptor interactions, T cell receptor signaling, and hematopoietic cell lineage, which is mostly comprised of T cell lineage markers (Additional file 10: Table S11D). Primary immunodeficiency was specifically inhibited (*p* = 0.0041), reflecting the up regulation of Cd8 (marker for cytotoxic T cells), Cd3δ, Cd3ε (required for differentiation of pro-T cells into pre-T cells) and Lck (required for Cd4^+^Cd8^+^ T cell differentiation into Cd8^+^ T cells) [58]. This is consistent with a strong Cd8+ T cell differentiation program in CPA-treated GL261(B6) tumors. Cell adhesion and antigen processing and presentation were also specifically up regulated in GL261(B6) compared to GL261(scid) tumors, consistent with the activation and contribution of Cd8^+^ T cells to tumor regression in regressing GL261(B6) tumors [20]. The top KEGG pathway enriched in genes specifically induced by CPA/6d in GL261(scid) tumors (Additional file 10: Table S11E), calcium signaling, may relate to the hyperactive innate immune system of scid mice [59].

Five UPRs showed a response to CPA treatment unique to either B6 or scid mice (Additional file 4: Table S3J, K). No inhibited UPRs were unique to either mouse model. Three activated UPRs associated with immune activation were unique to CPA/6d-treated GL261(B6) tumors: EIF2AK2 [26], IFNL1 [60] and MAVS [61], while the two activated UPRs specific to GL261(scid) tumor responses, DMD [62] and SPARC [34–36], have glial cell-related functions:.

### Gene responses associated with 6-day vs. 9-day metronomic CPA schedules

The metronomic CPA treatment schedule can have a major impact on the extent of immune cell infiltration into treated tumors and the overall effectiveness of the anti-tumor response [17, 18]. Significant NK cell recruitment and major tumor regression are achieved when GL261-bearing scid mice are given CPA on either a 6 day (CPA/6d) or a 9 day repeating schedule (CPA/9d); however, the NK cell response is transient on the CPA/9d schedule and is followed by early tumor growth rebound, whereas NK cell recruitment and tumor growth delay are both prolonged on the CPA/6d schedule [18]. We used these models to identify early molecular signaling events associated with these schedule-dependent NK cell and tumor regression responses by investigating gene expression changes 6 days after two CPA/6d or two CPA/9d treatment cycles, at which time major tumor regression is evident on both schedules [18].

Large numbers of genes were responsive to both CPA treatment schedules (Additional file 1: Table S1D-S1F), with many genes regulated in common (Additional file 1: Table S1F; Additional file 2: Figure S2). Many fewer genes showed differential regulation between the CPA/6d and CPA/9d schedules (Additional file 11: Table S12A, Additional file 2: Figure S10A). To identify genes specifically dysregulated by each CPA schedule, we relaxed the stringency filter to a >1.33-fold difference in response between schedules, while keeping the significance filter for genes under consideration unchanged at |FC| > 2 and *p* < 0.001 (Additional file 2: Figure S10B, Additional file 11: Table S12B). The set of 54 CPA/6d schedule-specific up regulated genes was enriched for cytokine-cytokine receptor interactions and other immune-related pathways (Additional file 12: Table S13A, B), suggesting that the CPA/6d schedule activates a more extensive immune response. The set of 172 genes preferentially up regulated on the CPA/9d schedule was enriched for histidine metabolism (Additional file 12: Table S13C) and for a C-type lectin cluster (Additional file 12: Table S13D). This cluster includes 3 inhibitory lectin receptors expressed on myeloid and/or NK cells (CLEC1A [63], KLRA1 [64], KLRA3 [65]), KLRA10, which is closely related in structure to inhibitory Ly49C receptors [66], and MGL2, a marker for pro-tumor macrophages [67]. These genes may contribute to the inability of the CPA/9d schedule to sustain an anti-tumor immune response.

Applying a |FC| > 1.5 and *p* < 0.001 gene expression filter to compare responses in CPA/9d-treated tumors with CPA/6d-treated tumors, we identified 151 genes expressed at a higher level and 10 genes expressed at a lower level in CPA/9d-treated tumors than in CPA/6d-treated GL261(scid) tumors (Additional file 11: Table S12C; Additional file 2: Figure S10C). The top enriched pathways in the 151 gene set, drug/xenobiotic metabolism and glutathione metabolism (Additional file 12: Table S13E), relate to chemotherapy resistance. Another enriched pathway, hematopoietic cell lineage, is associated with platelet, erythrocyte, neutrophil, and macrophage differentiation (Additional file 2: Figure S11), which may be linked to tissue repair and resistance to chemotherapy. Glycolysis/gluconeogenesis, which is also enriched among these 151 genes, might reflect increased tumor cell proliferation. The 10 genes showing lower expression in CPA/9d-treated tumors includes CXCL11, which suggests reduced anti-tumor IFN signaling [68].

Finally, we considered individual genes with an expression intensity (normalized RNA-seq read number) > 50 in either untreated or CPA-treated tumors, among the gene changes showing >2-fold differential response to each CPA schedule. PER2, TM7SF4, RETNLA and 10 other genes were thus identified as being uniquely up regulated on the CPA/9d schedule; no genes were uniquely down regulated by the CPA/9d schedule (Additional file 11: Table S12D). PER2 is a potential negative regulator of TLR9-mediated innate and adaptive immune responses [69]. TM7SF4 is a negative regulator of dendritic cell activity that can maintain immune self-tolerance [70], and RETNLA is highly expressed in alternative activated (M2) macrophages [71] and can stimulate proliferation of mesenchymal stem cells as well as angiogenesis [72]. Thus, these genes may contribute to the impaired NK cell response seen in CPA/9d-treated tumors. Further, XIST was uniquely up regulated – more than 20-fold – and SMPD3 was uniquely down regulated by the CPA/6d schedule (Additional file 11: Table S12D). XIST expression correlates with ovarian cancer cell sensitivity to taxol and high XIST levels are associated with late relapse [73]. SMPD3, which is down regulated uniquely in CPA/6d-treated GL261(scid) tumors, can promote angiogenesis within the tumor microenvironment as well as metastasis by regulating exosomal microRNA secretion [74]. No negative regulators of immune response were unique to either CPA schedule.

## Discussion

The immune anti-tumor action of CPA is highly dependent on schedule, as seen in several glioma models, where optimal immune-based regression is achieved using a 6-day repeating metronomic schedule of drug treatment (CPA/6d), but not when using more frequent [16, 17] or less frequent scheduling [18]. This ability of CPA/6d treatment to activate a strong, tumor regressing immune response is highly dependent on the tumor model, as shown by our studies of LLC Lewis lung carcinoma and B16F10 melanoma, which are intrinsically sensitive to activated CPA in cell culture but do not exhibit the potent CPA-inducible anti-tumor immune responses and robust regression seen with GL261 gliomas in the same syngeneic B6 mouse model. Global transcriptional profiling, by RNA-seq, was employed to identify the gene, pathway and upstream regulator (UPR) responses that underlie the differential CPA immune responsiveness of GL261 (responsive) versus LLC and B16F10 (unresponsive) tumors, and to elucidate the impact of host immune status and schedule of metronomic chemotherapy administration on these gene responses.

### Molecular mechanisms associated with differential tumor responsiveness to metronomic CPA

Immune stimulatory and immune effector KEGG pathways dominated the top up regulated gene responses to metronomic CPA treatment (CPA/6d schedule) in GL261, but not LLC or B16F10 tumors, while tumor cell survival pathways typified the down regulated gene responses. Consistently, a majority of immunogenic cell death pathway genes with good prognostic impact [75] were induced by CPA/6d treatment in GL261 tumors, but not in LLC or B16F10 tumors. These include genes involved in TLR signaling, the purinergic receptor-Inflammasome-interleukin1β axis, innate immune effectors, T cell infiltration, and T cell effectors. Examination of the UPRs linked to the gene responses induced by CPA in each tumor model revealed that many UPRs uniquely associated with CPA/6d-treated GL261 tumors are factors that facilitate tumor regression by immune mechanisms or inhibit tumor cell survival (Table 2). Few unique UPRs were associated with B16F10 tumors, and even fewer UPRs with LLC tumors, which showed the weakest anti-tumor response and the fewest dysregulated genes (Table 1), despite their high intrinsic sensitivity to CPA cytotoxicity. One UPR, the pro-angiogenic factor VEGFA, was activated by CPA treatment in GL261 tumors but was uniquely inhibited in LLC tumors, as was validated by analysis of CPA effects on LLC tumor microvessel density. This differential activation status of VEGFA between GL261 and LLC tumors correlates with the level of immune cell marker gene infiltration in each tumor model and is consistent with the requirement for VEGFA/VEGFR2 signaling for CPA to activate immune cell recruitment in responding gliomas [16, 19]. Both KEGG pathways down regulated in LLC tumors, cell adhesion and leukocyte transendothelial migration, are critical for immune cell recruitment into the tumor compartment. Presumably, the VEGFA inhibition/anti-angiogenesis seen in CPA/6d-treated LLC tumors combined with the down regulation of cell adhesion molecules and leukocyte transendothelial migration suppresses an important route of immune cell infiltration.

CPA/6d treatment of B16F10 tumors induced more prolonged growth delay and many more gene responses, including immune-related gene responses, than in LLC tumors (Table 1). The CPA/6d-treated B16F10 tumors shared 59 UPRs in common with GL261 tumors, including several UPRs important for immune cell responses, notably IFNAR1, IFNB1, IFNG, and IRF7. However, B16F10 tumor gene responses were not enriched for any of the immune effector KEGG pathways found in GL261 tumors, such as NK cell cytotoxicity and T cell receptor signaling, even though other immune activation pathways were enriched, including cytokine-cytokine receptor interaction, chemokine signaling pathway, and antigen processing and presentation. Direct comparison of the CPA-induced gene responses between responsive (GL261) and unresponsive (LLC and B16F10) tumor models (Additional file 13: Table S6) suggested that activation of T cell receptor signaling, inhibition of primary immunodeficiency, and leukocyte transendothelial migration are more extensive (greater fraction of pathway genes, lower *p*-value) in the responsive tumors (Additional file 13: Table S6F). CPA induced only four chemokines in B16F10 tumors, CCL5, CCL6, CCL7 and CCL9, all related to monocyte and lymphocyte recruitment [76], while in LLC tumors only one chemokine was induced, CCL11, which recruits eosinophils [77]. In contrast, 30 different chemokine and chemokine receptor genes, many important for NK cell and T cell recruitment, were up regulated in CPA-treated GL261 tumors (Additional file 1: Table S1A). Based on these findings, adoptive transfer of activated lymphocytes and/or delivery of chemokines that increase NK cell or T cell tumor infiltration might synergistically enhance responses to CPA treatment of B16F10 tumors.

### High basal immune infiltration in GL261 tumors

As not all tumors intrinsically sensitive to CPA cytotoxicity mount a strong immune response to metronomic CPA, there is a need for markers to phenotype individual tumors and select patients for CPA-based immunogenic chemotherapy. Markers of immunogenic tumor cell death alone are not likely to be sufficient, insofar as HMGB1 release occurs in CPA-treated B16F10 tumors [13], which we found are largely immune unresponsive to CPA/6d treatment. Indeed, basal levels of expression were similar or higher in the unresponsive tumors (LLC, B16F10) compared to the responsive (GL261) tumors for genes associated with immunogenic cell death danger signal formation (CALR, HMGB1, HSP90AA1) and immunogenic cell death execution (ATG5, BAX, CASP8, PDIA3, PIK3CA) (c.f. [75]). Further, immunogenic cell death pathway danger signal degradation factors (ENTPD1, NT5E) were the same or lower in the unresponsive tumors. We also found, however, that basal expression of many downstream immune stimulatory and immune-effector genes, including genes associated with T cell infiltration, is higher in the responsive compared to unresponsive tumor models (Additional file 8: Table S8A). The latter finding is consistent with the low level of MHC I expression reported in untreated LLC and B16F10 (i.e., unresponsive) tumors in B6 mice [78], and suggests that basal tumor immunogenicity may be predictive of a CPA/6d-inducible immune response, in accordance with the correlation between tumor immunogenicity and efficacy of immunotherapy [78]. Of note, GL261 tumors are considered moderately immunogenic [79]. Interestingly, genes showing higher basal expression in GL261 tumors were significantly enriched for CPA-responsive genes, suggesting that CPA/6d treatment activates pathway(s) that are already primed to be differentially expressed between responsive and unresponsive tumors. Basal gene expression profiles may thus be useful in identifying tumors, and perhaps patients, more likely to show effective immune responsiveness to metronomic CPA. Studies in additional tumor models, including gliomas with lower immunogenicity than GL261, are needed to confirm and extend these findings and to exclude confounding factors such as differences in mutation status and tumor histology. Histological differences between the responsive (glioma) and unresponsive (lung carcinoma, melanoma) tumors examined are probably not a major factor in their differential CPA immune responsiveness, as indicated by the rather small number of neuronal-related KEGG pathways and UPRs linked to the GL261-specific gene responses (Additional file 8: Table S8A, Table 2).

### Negative regulators of immune response

An immune suppressive tumor microenvironment may dampen the efficacy of chemotherapy [80] and could be a factor in the immune unresponsiveness of LLC and B16F10 tumors. Three KEGG pathways with immune suppressive potential were enriched in the gene set expressed at a lower level in CPA-treated GL261 than LLC and B16F10 tumors: steroid biosynthesis, which can inhibit T cell development [81]; butanoate metabolism, which can induce T cell apoptosis [82]; and tryptophan metabolism, which can induce regulatory T cell proliferation [83] (Additional file 14: Table S9C), suggesting the unresponsive tumors have a more highly immune suppressive environment. However, many negative regulators of immune response actually showed *higher* basal expression in GL261 tumors, and/or were more commonly up regulated by CPA treatment in GL261 compared to LLC and B16F10 tumors. This latter finding is consistent with a feedback response leading to up regulation of immunosuppressive T regulatory cells in CPA-treated GL261 tumors [20, 84]. This effect is minimized by giving CPA on a 6 day schedule [20], and may be a factor driving the need for repeated CPA treatment to maximize anti-tumor immune activity.

### Impact of scid immunodeficiency

CPA/6d treatment of GL261 tumors implanted in adaptive immune deficient scid mice induces major regression, but is sometimes followed by late tumor growth rebound [18], whereas in the fully immune competent B6 mouse model, GL261 tumors are eradicated by a Cd8 T cell-dependent mechanism with acquisition of long term immunity [20]. A subset of cytokine-cytokine receptor interaction, T cell receptor signaling and several other immune-related KEGG pathway genes were unique to the GL261(B6) tumor model as compared to the GL261(scid) model, consistent with the importance of T cell receptor signaling and cytokine-cytokine receptor interaction for the more complete and long-lasting anti-tumor responses in GL261(B6) tumors [20]. Further, three activated UPRs associated with immune activation were unique to CPA/6d-treated GL261(B6) tumors: EIF2AK2 is an intracellular danger-sensing molecule important for inflammasome activation and HMGB1 release [26]; IFNL1 can mediate inflammatory responses in epithelial cells [60, 85]; and MAVS is required for activation of NFkB and interferon induction in response to viral infection [61]. The two activated UPRs specific to GL261(scid) tumor responses have glial cell-related functions: DMD may relate to glial cell differentiation [62]; and SPARC can inhibit brain tumor cell growth but also promotes invasion by increasing binding of extracellular matrix proteins [34–36].

### Extending CPA drug-free break from 6 to 9 days

We investigated gene responses in GL261(scid) tumors treated with CPA on a 9 day repeating schedule (CPA/9d), which initially induces tumor regression nearly as effectively as CPA/6d treatment, but leads to a striking rebound in tumor growth after treatment day ~24, despite ongoing CPA treatment [18]. We found only a small number of significant gene response differences between the two CPA schedules, consistent with the very similar overall tumor regression status of these two tumor models at the time of tumor sampling after two CPA treatment cycles. Genes specifically up regulated on the CPA/6d schedule were enriched in cytokine-cytokine receptor and other immune-related pathways, consistent with the more sustained NK cell response in CPA/6d-treated GL261(scid) tumors [18], while the CPA/9d schedule preferentially up regulated a C-type lectin gene cluster that includes several inhibitory lectin receptors expressed on myeloid and/or NK cells (CLEC1A [63], KLRA1 [64], and KLRA3 [65]). Other top pathways preferentially enriched in CPA/9d-treated tumors include drug metabolism and glutathione metabolism, which can confer chemotherapy resistance, suggesting that the longer drug-free breaks on the CPA/9d schedule facilitate selection of drug resistant tumor cells.

## Conclusion

We identified several factors that are associated with, and may contribute to the strong immune responsiveness of GL261 tumors to a 6 day repeating metronomic schedule of CPA treatment; these include elevated basal levels and higher CPA-induced levels of many immune factors, enhanced blood vessel morphogenesis and leukocyte transendothelial migration, and reduced basal expression of drug metabolism genes compared to LLC and/or B16F10 tumors. Further studies, including genetic ablation or pharmacological inhibition of key factors linked to immune responsiveness, are required to establish causal roles for these factors. Furthermore, analysis of isolated tumor cell and immune cell populations may help elucidate the cellular origin of these immune response regulators. These factors likely work in concert to increase immunogenic responses to metronomic CPA scheduling, and when taken together, may help identify individual tumors most likely to be responsive to immunogenic chemotherapy. This approach is complementary to ongoing efforts to characterize tumors by an immunoscore that integrates immune cell type, density, location, and functional state and predicts cancer patient responsiveness to therapy [86].

## Abbreviations

B6, C57BL/6 mouse strain; CPA, cyclophosphamide; CPA/6d or CPA/9d, metronomic CPA scheduling at 140 mg CPA/kg body weight, repeated every 6 days or every 9 days, respectively; GL261(B6), GL261 tumors implanted in B6 mice; GL261(scid), GL261 tumors implanted in scid mice; IPA, Ingenuity Pathway Analysis; KEGG, Kyoto Encyclopedia of Genes and Genomes; LLC, Lewis lung carcinoma; NK cell, natural killer cell; RNA-seq, RNA-based sequencing; scid, severe combined immunodeficiency; UPR, upstream regulator

## Additional files

Additional file 1: Table S1. Significant gene responses induced by metronomic CPA treatment in each of the indicated tumor models. Shown in Tables S1A-S1F are genes differentially expressed between CPA-treated and control (drug-free) GL261(B6) tumors (Table S1A), LLC tumors (Table S1B), B16F10 tumors (Table S1C), and GL261(scid) tumors (Table S1D, following treatment with CPA/6d; Table S1E, treatment with CPA/9d; and Table S1F, genes common to CPA/6d and CPA/6d treatment). Treatments and other data about RNA-seq samples, including GSM accession numbers within GEO data set GSE71491, are also shown. (XLSX 3150 kb) Additional file 2: Figure S1-S11. Figure S1. Venn diagrams showing the numbers of genes that responded to CPA treatment. Figure S2. UPR analysis comparing GL261(B6) tumors vs. LLC and B16F10 tumors, and comparing GL261(B6) tumors vs. GL261(scid) tumors. Figure S3. Strategy for identification of stringent UPRs. Figure S4. Identification of stringent UPRs that are unique to one tumor model as compared to a second tumor model. Figure S5. Identification of stringent gene responses to CPA treatment in GL261(B6) tumors compared to B16F10 and LLC tumors. Figure S6. Identification of gene responses to CPA in GL261(B6) compared to GL261(scid) tumors. Figure S7. Differential basal gene expression in GL261(B6) vs. LLC and B16F10 tumors. Figure S8. Negative regulators of immune responses in untreated or CPA-treated GL261(B6), LLC and B16F10 tumors. Figure S9. Differential gene expression comparing CPA-treated GL261(B6) vs. LLC and B16F10 tumors. Figure S10. Differential gene responses to CPA/6d and CPA/9d schedules in GL261(scid) tumors. Figure S11. Hematopoietic cell lineage KEGG pathway. This pathway is enriched in genes expressed >1.5-fold higher in GL261(scid) tumors treated on a CPA/9d schedule than in tumors treated on a CPA/6d schedule. (PDF 1.66 mb) Additional file 3: Table S2. Listed are all CPA-induced upstream regulators (UPRs) as predicted by IPA for CPA-treated GL261(B6) (Table S2A), LLC (Table S2B), B16F10 (Table S2C), GL261(scid) (Table S2D; CPA/6d treatment), and GL261(scid) (Table S2E; CPA/9d treatment) tumors, respectively. (XLSX 960 kb) Additional file 4: Table S3. Listing of stringent UPRs induced by CPA treatment in GL261(B6) (Table S3A), LLC (Table S3B), B16F10 (Table S3C), GL261(scid) (Table S3D; CPA/6d treatment), GL261(scid) (Table S3E; CPA/9d treatment), and GL261(scid) tumors (Table S3F; based on genes common to CPA/6d and CPA/9d treatment). (XLSX 287 kb) Additional file 5: Table S4. KEGG pathways enriched in genes up regulated or down regulated by CPA treatment in GL261(B6) tumors (Table S4A), LLC tumors (Table S4B), and B16F10 tumors (Table S4C). (XLSX 32 kb) Additional file 6: Table S5. Negative regulators of immune response. (XLSX 70 kb) Additional file 7: Table S7. Genes whose basal expression in untreated GL261(B6) tumors is either higher or lower than in untreated LLC and untreated B16F10 tumors. (XLSX 670 kb) Additional file 8: Table S8A-F. KEGG pathways and DAVID clusters enriched in genes that are specifically expressed at a higher or a lower level in untreated GL261(B6) tumors when compared to LLC and B16F10 tumors and in the sets of genes that respond to CPA/6d treatment. (XLSX 186 kb) Additional file 9: Table S10. Differential gene responses to CPA in GL261(scid) and GL261(B6) tumors. (XLSX 2296 kb) Additional file 10: Table S11. KEGG pathways enriched in genes commonly or differentially expressed in GL261(B6) and GL261(scid) tumors in response to CPA treatment. (XLSX 32 kb) Additional file 11: Table S12. Differential gene responses in GL261(scid) tumors induced by CPA/9d or CPA/6d treatment. (XLSX 452 kb) Additional file 12: Table S13. KEGG pathways and DAVID clusters that are associated with genes preferentially up regulated (>1.33-fold) in GL261(scid) tumors treated with CPA/6d (Table S13A, B) or CPA/9d (Table S13C, D). Also shown (Table S13E) are KEGG pathways enriched in the set of genes expressed at a level 1.5-fold higher level in GL261(scid) tumors treated on the CPA/9d schedule than those treated on CPA/6d schedule. (XLSX 55 kb) Additional file 13: Table S6. Responses to metronomic CPA in responsive tumors compared to unresponsive tumor models. Shown are the differential gene responses, and associated KEGG pathways or DAVID clusters, induced by CPA/6d treatment of GL261(B6) tumors compared to LLC and B16F10 tumors. (XLSX 718 kb) Additional file 14: Table S9. Gene pathways predictive of treatment prognosis in CPA-treated tumors - We investigated whether gene pathways that may predict treatment outcome can be identified by comparing CPA-treated responsive vs. CPA-treated unresponsive tumors. (XLSX 634 kb)
