# Supplementary material for: Metronomic cyclophosphamide activation of anti-tumor immunity: tumor model, mouse host, and drug schedule dependence of gene responses and their upstream regulators
Source: BMC Cancer. 2016 Aug 11;16:623. doi: 10.1186/s12885-016-2597-2 (PMC4982114; doi:10.1186/s12885-016-2597-2)
Supplement: Additional file 2: Figure S1-S11. — Figure S1. Venn diagrams showing the numbers of genes that responded to CPA treatment. Figure S2. UPR analysis comparing GL261(B6) tumors vs. LLC and B16F10 tumors, and comparing GL261(B6) tumors vs. GL261(scid) tumors. Figure S3. Strategy for identification of stringent UPRs. Figure S4. Identification of stringent UPRs that are unique to one tumor model as compared to a second tumor model. Figure S5. Identification of stringent gene responses to CPA treatment in GL261(B6) tumors compared to B16F10 and LLC tumors. Figure S6. Identification of gene responses to CPA in GL261(B6) compared to GL261(scid) tumors. Figure S7. Differential basal gene expression in GL261(B6) vs. LLC and B16F10 tumors. Figure S8. Negative regulators of immune responses in untreated or CPA-treated GL261(B6), LLC and B16F10 tumors. Figure S9. Differential gene expression comparing CPA-treated GL261(B6) vs. LLC and B16F10 tumors. Figure S10. Differential gene responses to CPA/6d and CPA/9d schedules in GL261(scid) tumors. Figure S11. Hematopoietic cell lineage KEGG pathway. This pathway is enriched in genes expressed >1.5-fold higher in GL261(scid) tumors treated on a CPA/9d schedule than in tumors treated on a CPA/6d schedule. (PDF 1.66 mb) [file 12885_2016_2597_MOESM2_ESM.pdf]

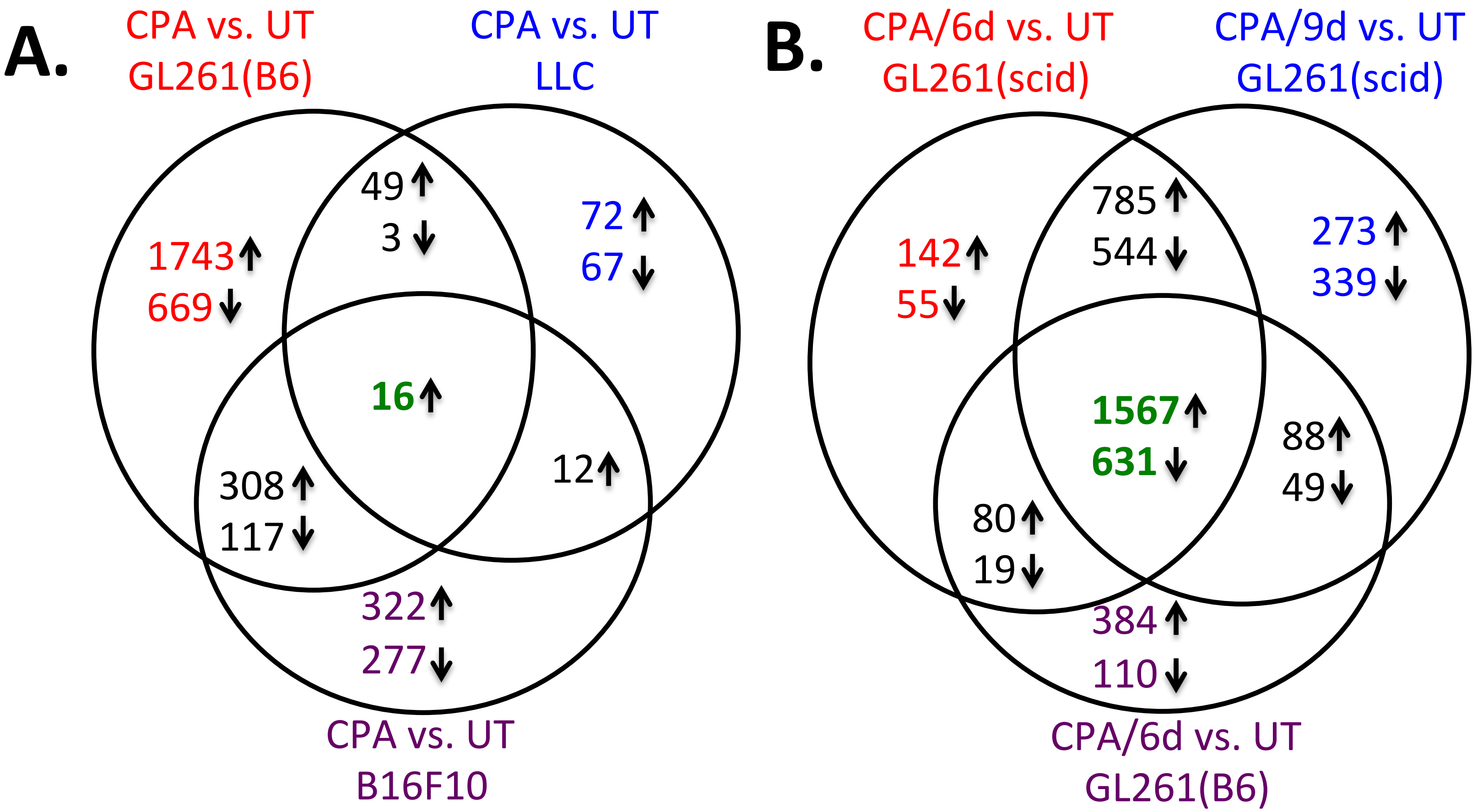

Fig. S1. **Venn diagrams showing the numbers of genes that responded to CPA treatment**. (A) GL261(B6), LLC, and B16F10 tumors treated with CPA/6d schedule; (B) GL261(scid) tumors treated with CPA/6d or CPA/9d schedule, and GL261(B6) tumors treated on CPA/6d schedule. Significance cut off values: |FC|>2, p<0.001. Shown are the number of genes that respond to CPA treatment, with up and down arrows indicating up and down regulation by CPA treatment, respectively. These gene numbers were not subject to a stringency filter based on fold changes between the different tumor models being compared. See Table S1 for full gene lists.

Fig. S2. **UPR analysis comparing GL261(B6) tumors vs. LLC and B16F10 tumors (*left*), and comparing GL261(B6) tumors vs. GL261(scid) tumors (*right*).**

Shown are the numbers of genes that respond to CPA treatment at |FC|>2 and p<0.001 by up regulation (red arrow) or down regulation (green arrow). These gene sets were uploaded to IPA to obtain the indicated numbers of Total UPRs identified (red boxes), which were filtered to obtain the indicated numbers of Stringent UPRs (as defined in Fig. S2; green boxes). Blue up arrows indicate activated UPRs and pink down arrows indicate inhibited UPRs. Significant genes or stringent UPRs that show common regulation (i.e., regulation in the same direction: up or down, activated or inhibited) are shown in blue boxes, and was determined by the comparisons of two sets parental genes or UPRs, marked by blue lines. The tables and supplementary tables listing the data sets used in these analyses are indicated.

See Fig. S4 for the strategy used to identify Stringent UPRs that are unique to one tumor model as compared to a second tumor model. As outlined here, comparison of Stringent UPRs in one tumor model (solid purple line) with Total UPRs identified in one (or two) other tumor model(s) (dashed purple lines) yielded a set of UPRs unique to one tumor model (dashed black boxes). For example, UPR analysis identified 150 activated and 29 inhibited stringent UPRs in common between CPA/6d-treated and CPA/9d-treated GL261(scid) tumors (Table S3F). 110 of these stringent UPRs (98 activated UPRs and 12 inhibited UPRs; Table S3I) are in common with the 145 activated and 35 inhibited stringent UPRs (Table S3A) identified in GL261(B6) tumors. Only 2 of the 150 activated and 29 inhibited stringent UPRs in common between CPA/6d-treated and CPA/9d-treated GL261(scid) tumors are unique UPRs in CPA-treated GL261(scid) tumors compared to CPA-treated GL261(B6) tumors, as determined by comparison to the full set of 2,121 Total UPRs identified in the GL261(B6) tumors.

Fig. S2. UPR analysis comparing GL261(B6) tumors vs. LLC and B16F10 tumors (*left*), and comparing GL261(B6) tumors vs. GL261(scid) tumors (*right*).

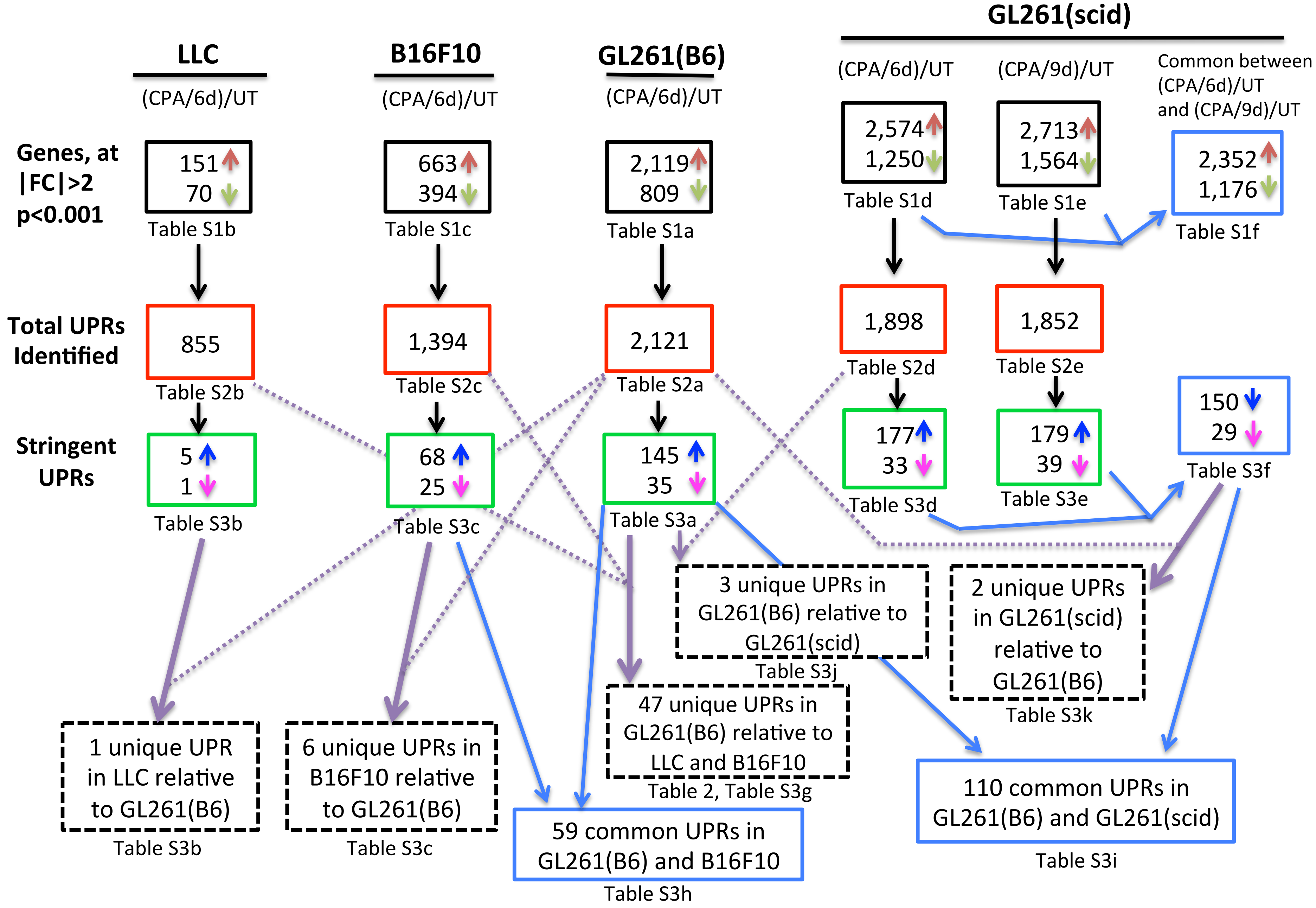

Fig. S3

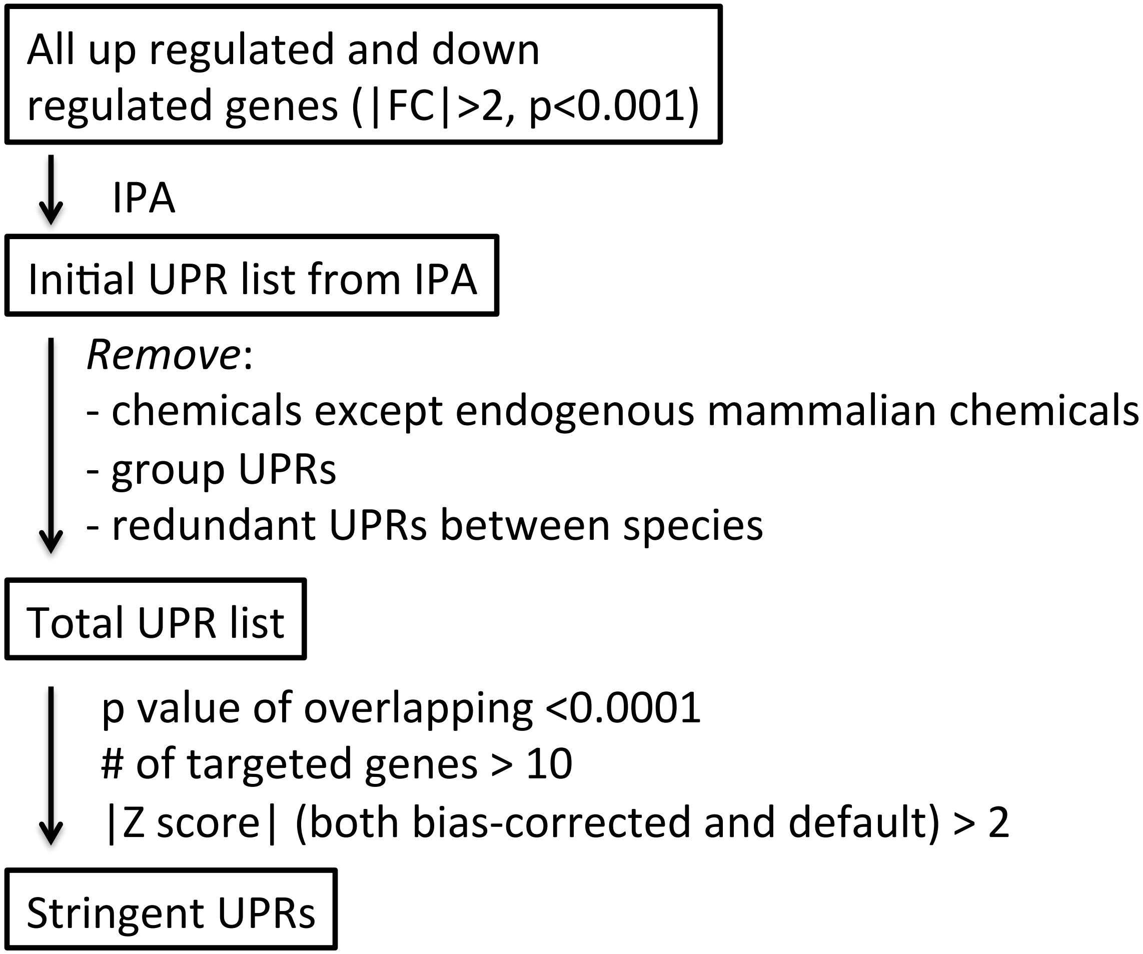

Fig. S3. **Strategy for identification of stringent UPRs**. A combined list of all genes up regulated or down regulated by CPA at |FC|>2 and p<0.001 was uploaded to IPA for UPR identification. The initial UPR list obtained from IPA was filtered to remove: UPRs that are chemicals, except endogenous mammalian chemicals, group UPRs, and redundant UPRs derived from two species (the UPR with the higher Z-score is retained). Further, UPRs that have p-value of overlap <0.0001, # of targeted genes > 10, and |Z score| (both bias-corrected and default) > 2 are defined as Stringent UPRs.

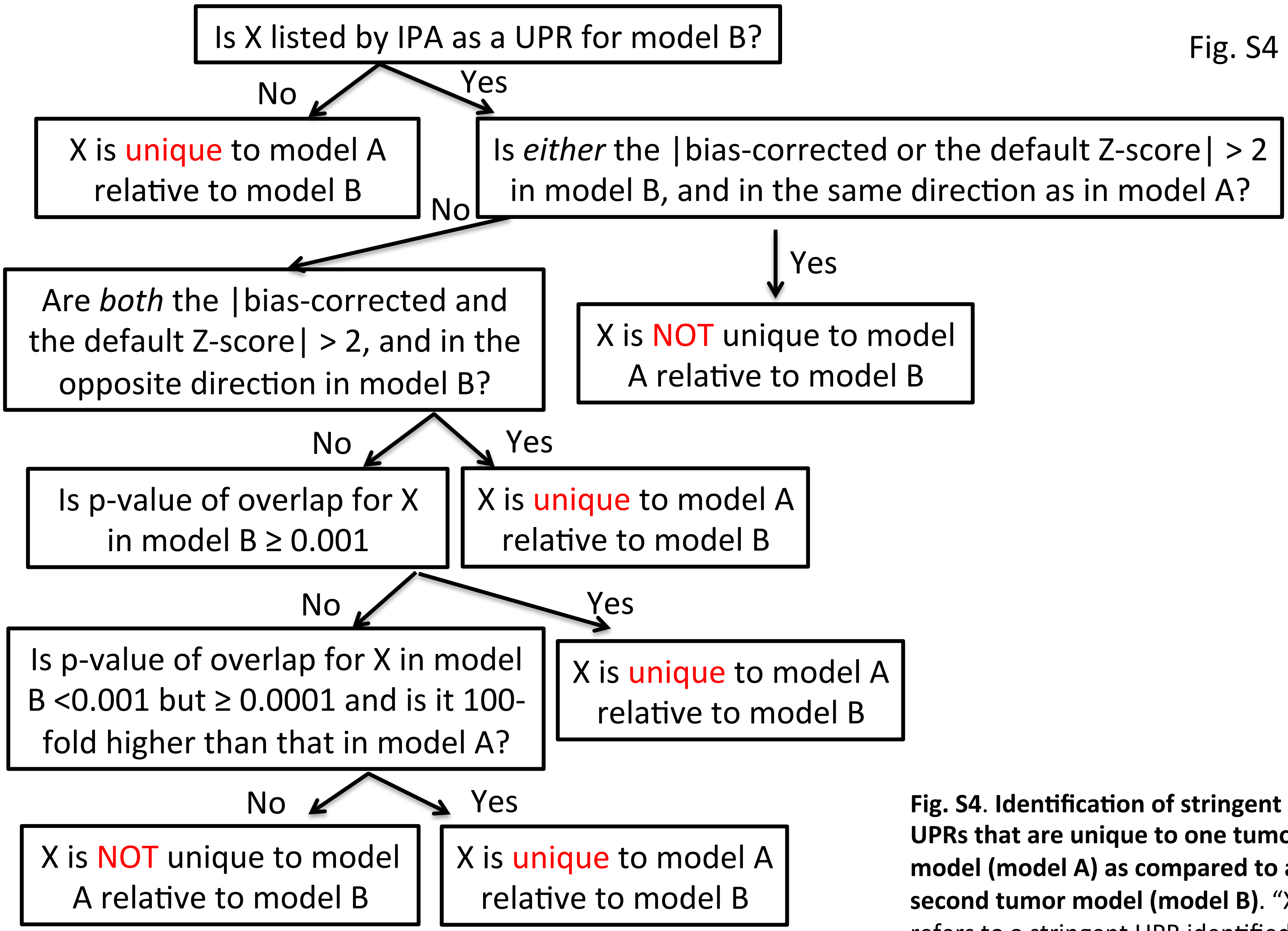

Fig. S4

Fig. S4. Identification of stringent UPRs that are unique to one tumor model (model A) as compared to a second tumor model (model B). "X" refers to a stringent UPR identified in model A (see Fig. S3)

Fig. S5

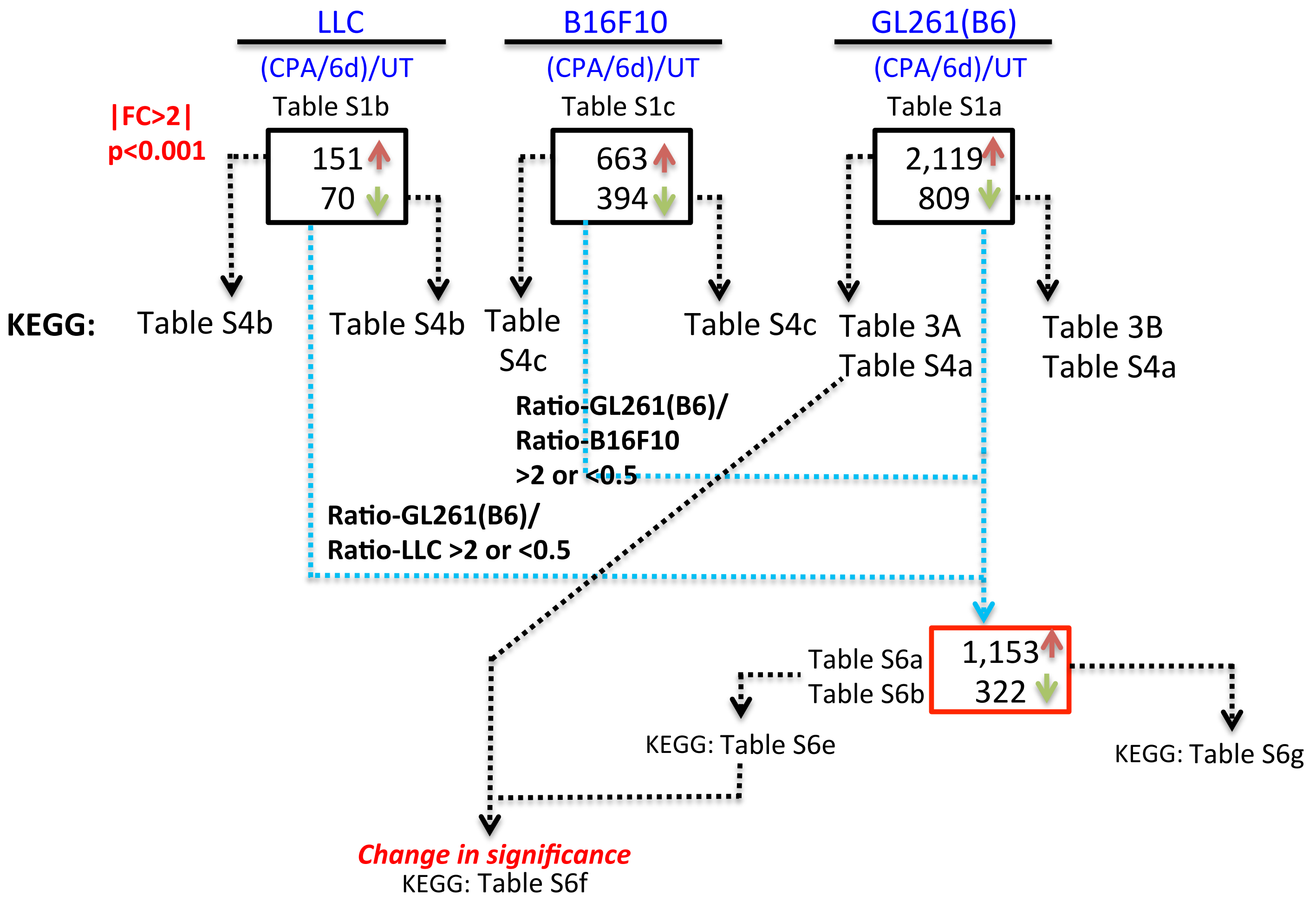

Fig. S5. Identification of stringent gene responses to CPA treatment in GL261(B6) tumors compared to B16F10 and LLC tumors. Genes up regulated (red arrow) and down regulated (green arrow) by CPA in GL261(B6) tumors (black boxes) were compared to the corresponding gene sets in B16F16 tumors to identify genes whose [Ratio-GL261(B6)/ Ratio-B16F10] >2 or <0.5. These genes were further compared to those identified in LLC tumors; genes that further met the condition [Ratio-GL261(B6)/Ratio-LLC] >2 or <0.5 were retained. The result is a gene set that is either unique or stringent in its response to CPA in GL261(B6) tumors compared to both B16F10 and LLC tumors (red box). Changes in relative significance between tumor models for the KEGG pathways identified are shown in Table S6F. The tables and supplementary tables that contain the corresponding gene sets and their associated KEGG pathways are indicated.

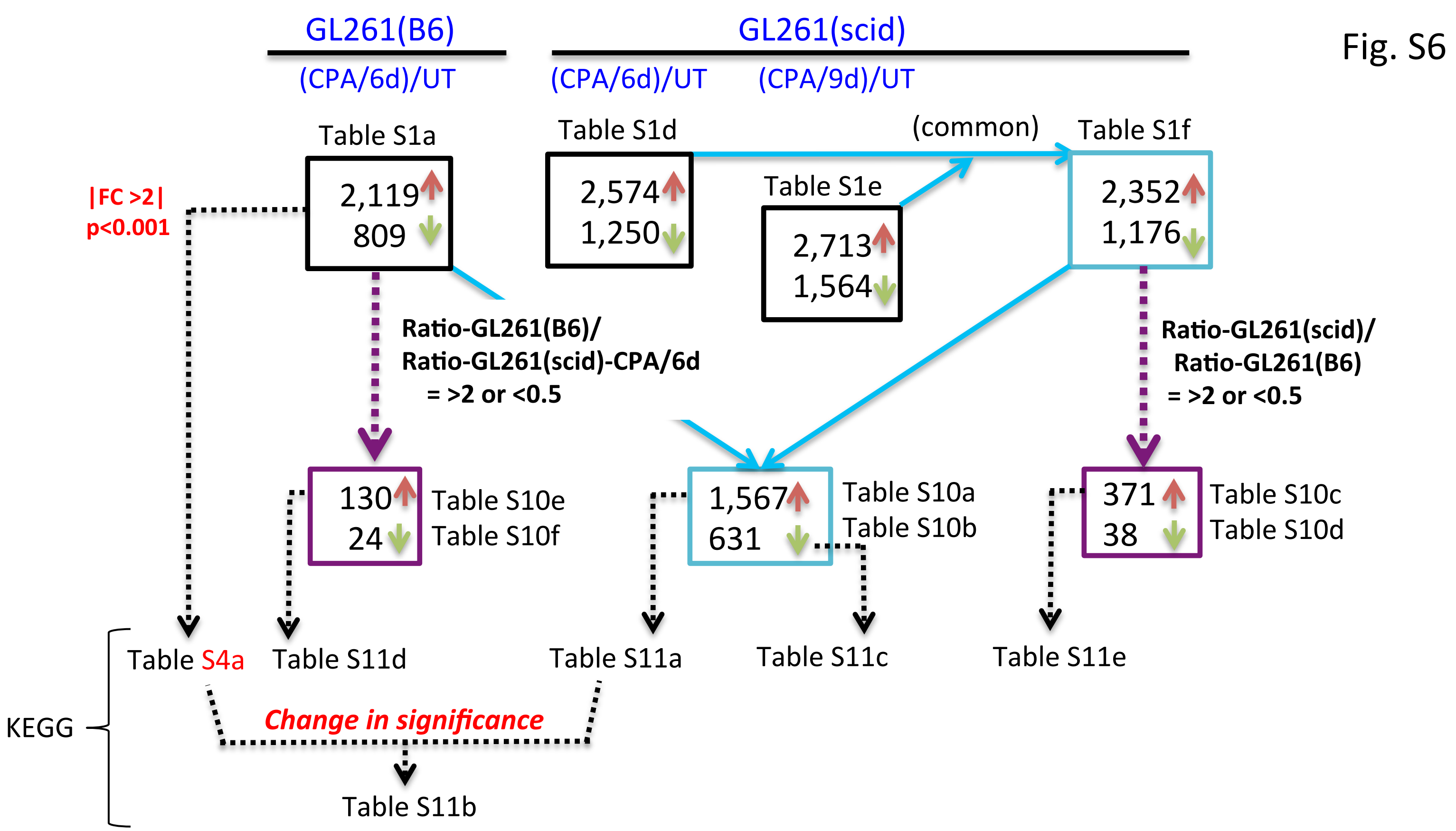

Fig. S6. Identification of gene responses to CPA in GL261(B6) compared to GL261(scid) tumors. Shown in the first row of boxes are the numbers of genes up regulated or down regulated by CPA in each of the tumor models indicated at top, or genes regulated in common in GL261(scid) tumors following CPA/6d or CPA/9d treatment (blue box, top right). The genes regulated in common between CPA/6d-treated and CPA/9d-treated GL261(scid) tumors were filtered to remove genes not showing at least 2-fold greater response to CPA than in GL261 (B6) tumors, yielding 371 up regulated genes and 38 down regulated genes specific to the GL261(scid) tumor response (purple box, *right*). We also filtered the genes responding to CPA/6d in GL261(B6) tumors to remove genes not showing at least 2-fold greater CPA response than in CPA/6d-treated GL261(scid) tumors, yielding 130 up regulated and 24 down regulated genes specific to the GL261(B6) tumor responses (purple box, *left*). Further, common responses to CPA in all three tumor models were seen for 1,567 up regulated genes and 631 down regulated genes (blue box, *middle*). Up-regulation, red arrows; down-regulation, green arrows. Blue lines: comparison of two datasets identifies a common dataset, shown in a blue rectangle. Purple dashed lines: comparison of two datasets identifies a dataset stringently associated with one dataset, shown in a purple rectangle. The tables and supplementary tables that contain the corresponding data sets are indicated. Changes in relative significance between tumor models for the KEGG pathways identified in GL261(B6) compared to GL261(scid) tumors are presented in Table S11B.

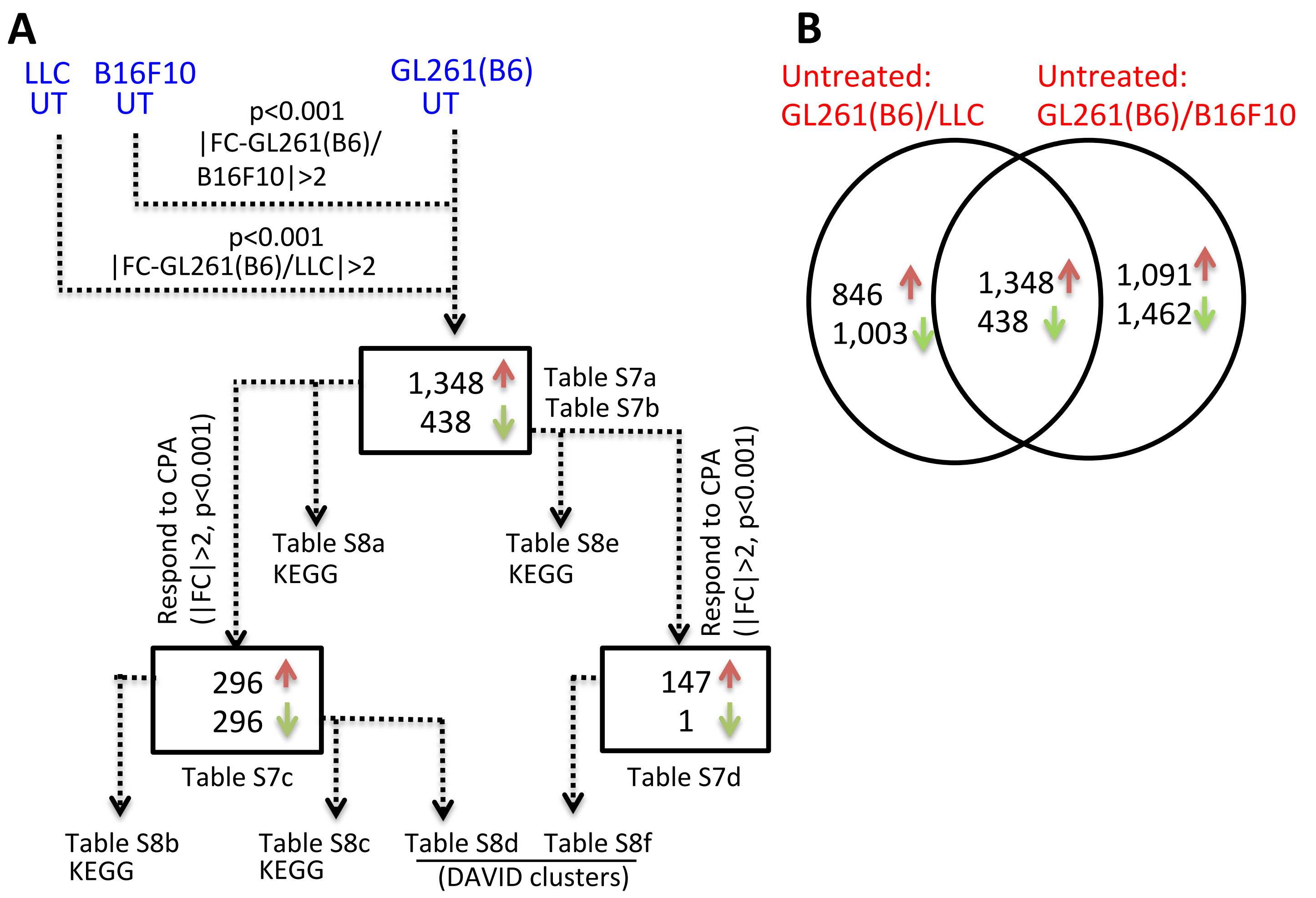

Fig. S7. Differential basal gene expression in GL261(B6) vs. LLC and B16F10 tumors.

Fig. S8

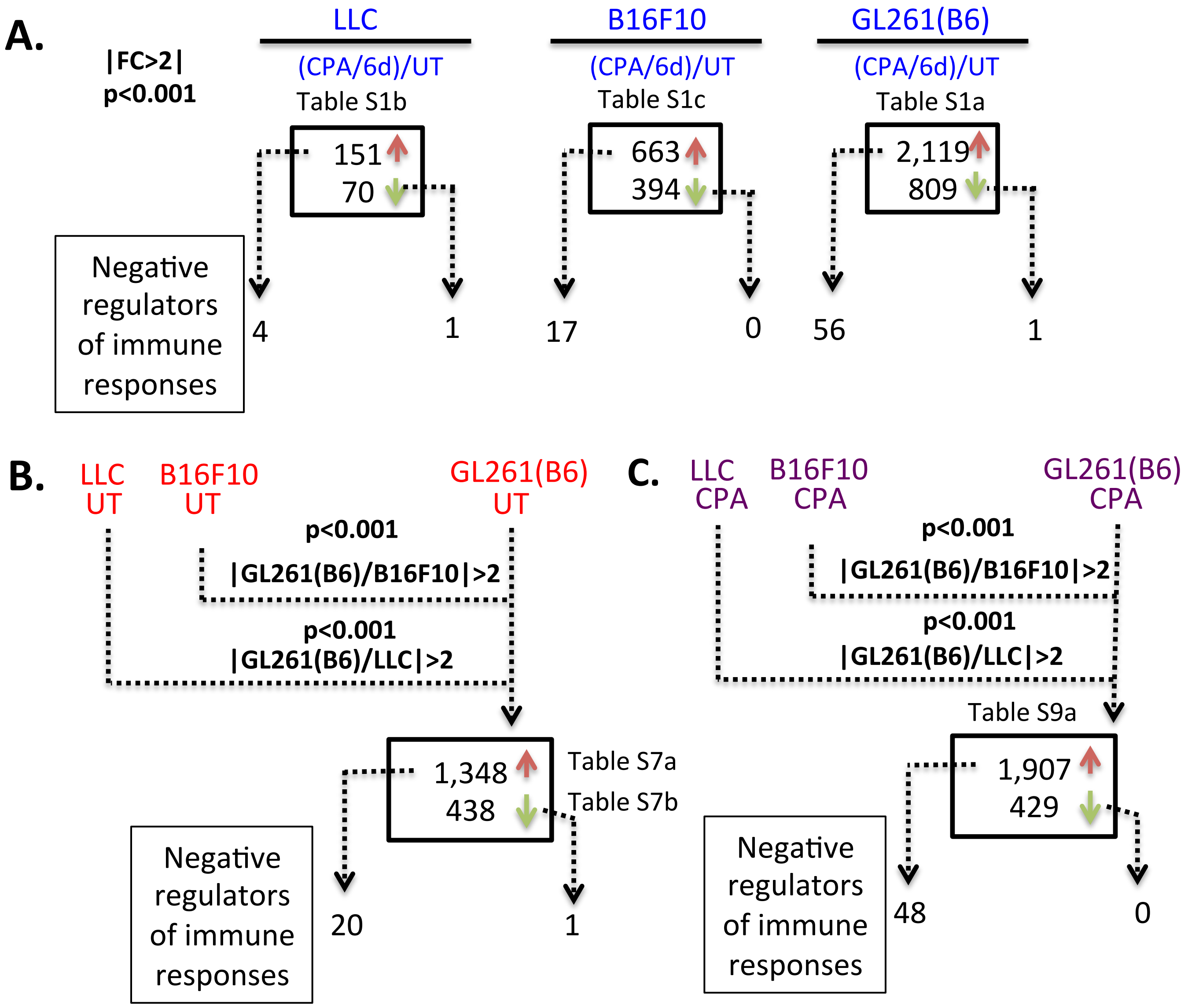

Fig. S8. Negative regulators of immune responses in untreated or CPA-treated GL261(B6), LLC and B16F10 tumors. (A) CPA-responsiveness; (B) Untreated tumors; (C) CPA-treated tumors. Significance cutoff values: |FC>2|, p<0.001.

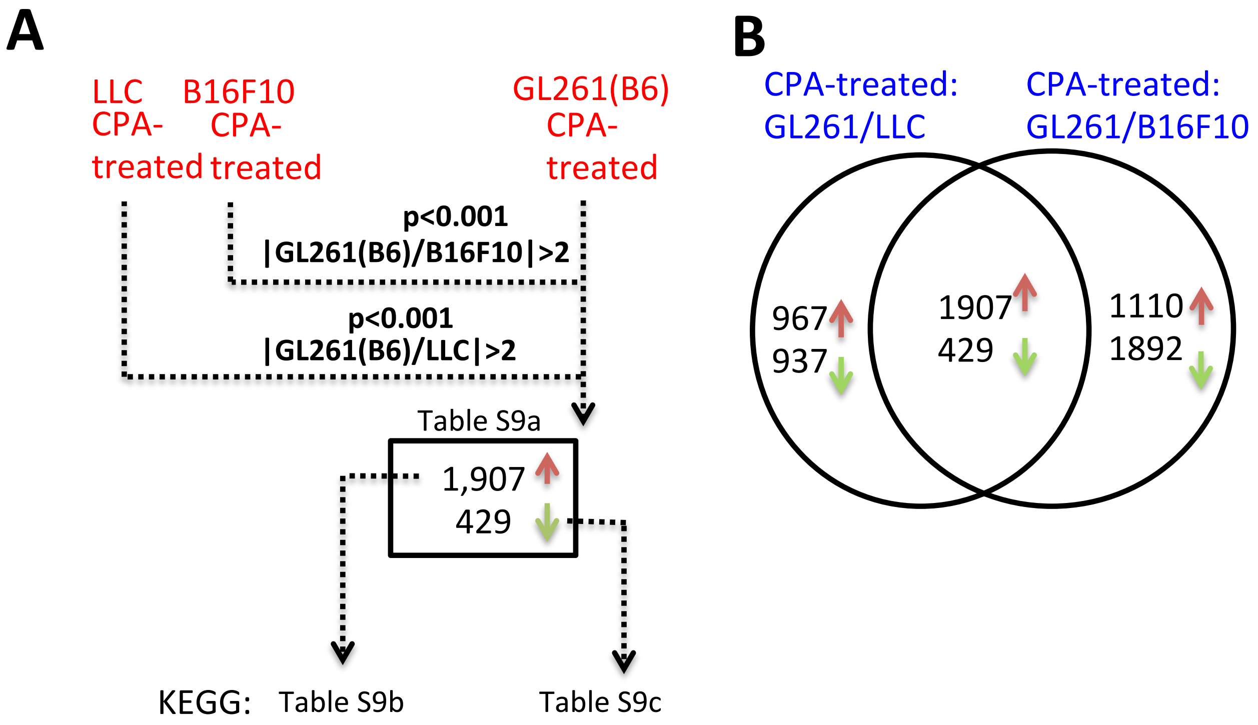

Fig. S9. **Differential gene expression comparing CPA-treated GL261(B6) vs. LLC and B16F10 tumors**. (A) data analysis; (B) Venn diagram showing differential expression pattern.

Fig. S10

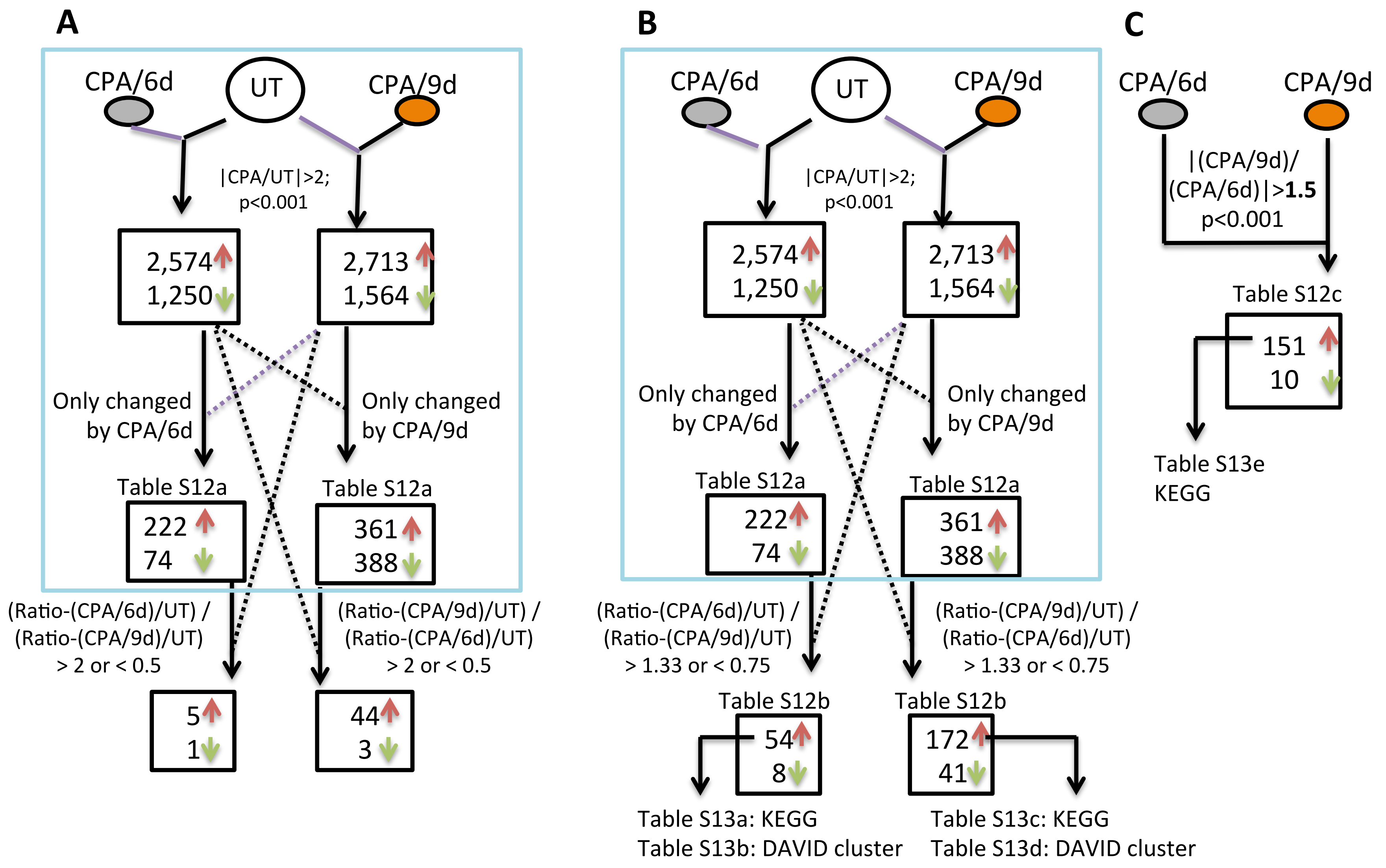

Fig. S10. Differential gene responses to CPA/6d and CPA/9d schedules in GL261(scid) tumors. (A) 2-fold change filter; Blue square region is same as in B. (B) 1.33-fold change filter; (C) Direct comparison between CPA/9d-treated and CPA/6d-treated tumors. UT, untreated tumors (drug-free controls).

Hematopoietic cell lineage

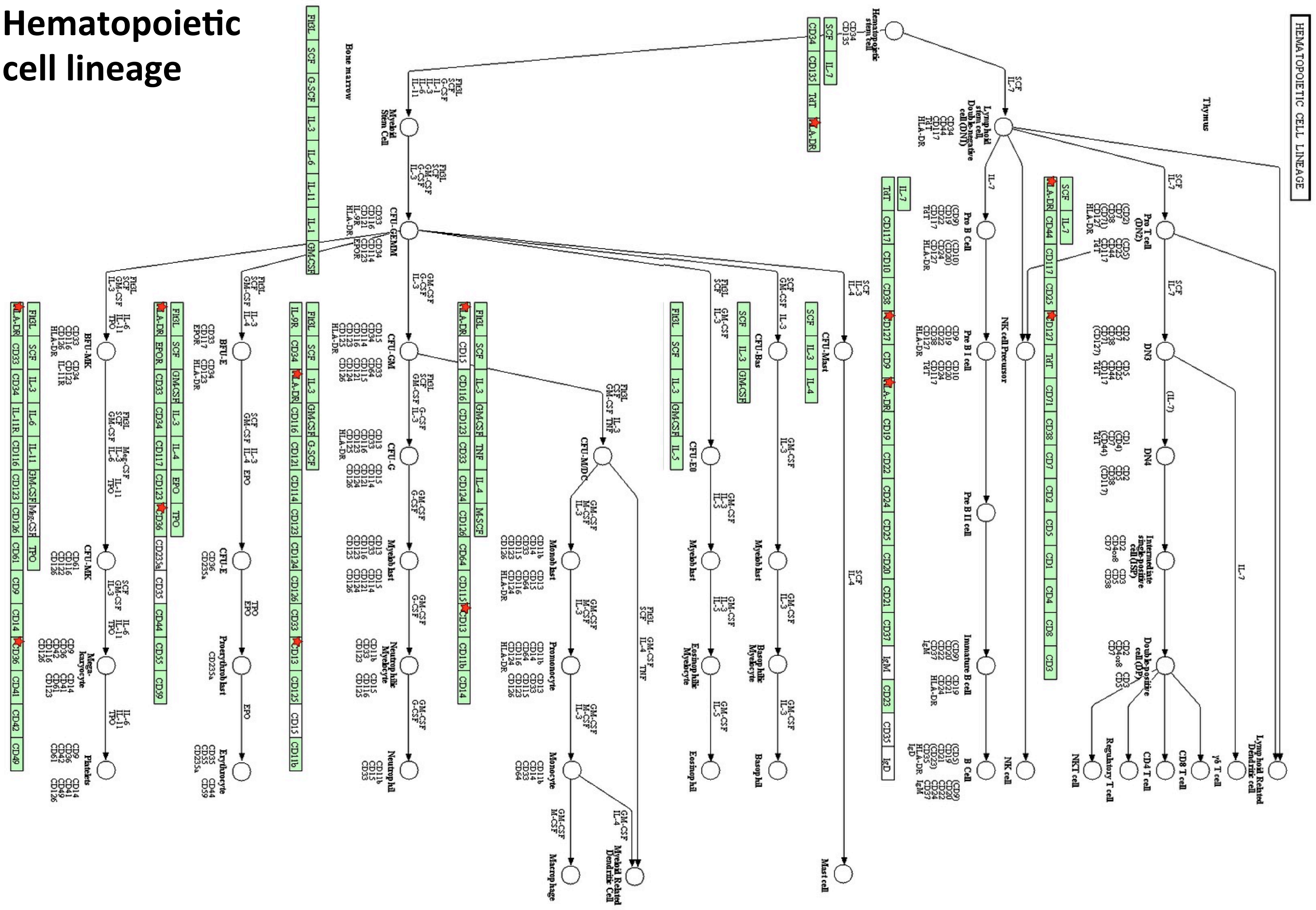

Fig. S11. **Hematopoietic cell lineage KEGG pathway**. This pathway (Table S13F) is enriched in genes expressed >1.5-fold higher in GL261(scid) tumors treated on a CPA/9d schedule than in tumors treated on a CPA/6d schedule. Matched genes in the pathway are labeled with a red star.
